# Targeting therapy and tumor microenvironment remodeling of triple-negative breast cancer by ginsenoside Rg3 based liposomes

**DOI:** 10.1186/s12951-022-01623-2

**Published:** 2022-09-15

**Authors:** Jiaxuan Xia, Shuya Zhang, Ru Zhang, Anni Wang, Ying Zhu, Meichen Dong, Shaojie Ma, Chao Hong, Shengyao Liu, Dan Wang, Jianxin Wang

**Affiliations:** 1 Department of Pharmaceutics, School of Pharmacy, Fudan University and Key Laboratory of Smart Drug Delivery, Ministry of Education, Shanghai, 201203 China; 2 Department of Integrative Oncology, Fudan University Shanghai Cancer Center, Shanghai, 200032 China; 3 Key Laboratory of Molecular Biophysics of the Ministry of Education, College of Life Science and Technology, Huazhong University of Science and Technology, Wuhan, 430071 China; 4 Experiment Center of Teaching and Learning, Shanghai University of Traditional Chinese Medicine, Shanghai, 201203 China; 5 Xiamen Ginposome Pharmatech Co., Ltd, Xiamen, 361026 People’s Republic of China; 6 Institutes of Integrative Medicine, Fudan University, Shanghai, 201203 People’s Republic of China

**Keywords:** Triple-negative breast cancer, Ginsenoside Rg3, Liposomes, Docetaxel, Tumor active targeting, Stroma cells, Cancer-associated fibroblasts, Tumor microenvironment

## Abstract

The chemotherapy effect of docetaxel (DTX) against triple-negative breast cancer (TNBC) remains mediocre and limited when encapsulated in conventional cholesterol liposomes, mainly ascribed to poor penetration and immunosuppressive tumor microenvironment (TME) caused by tumor stroma cells, especially cancer-associated fibroblasts (CAFs). Many studies have attempted to address these problems but trapped into the common dilemma of excessively complicated formulation strategies at the expense of druggability as well as clinical translational feasibility. To better address the discrepancy, ginsenoside Rg3 was utilized to substitute cholesterol to develop a multifunctional DTX-loaded Rg3 liposome (Rg3-Lp/DTX). The obtained Rg3-Lp/DTX was proved to be preferentially uptake by 4T1 cells and accumulate more at tumor site via the interaction between the glycosyl moiety of Rg3 exposed on liposome surface and glucose transporter1 (Glut1) overexpressed on tumor cells. After reaching tumor site, Rg3 was shown to reverse the activated CAFs to the resting stage and attenuate the dense stroma barrier by suppressing secretion of TGF-*β* from tumor cells and regulating TGF-*β*/Smad signaling. Therefore, reduced levels of CAFs and collagens were found in TME after incorporation of Rg3, inducing enhanced penetration of Rg3-Lp/DTX in the tumor and reversed immune system which can detect and neutralize tumor cells. Compared with wooden cholesterol liposomes, the smart and versatile Rg3-Lp/DTX could significantly improve the anti-tumor effect of DTX, providing a promising approach for TNBC therapy with excellent therapeutic efficacy and simple preparation process.

**Supplementary Information:**

The online version contains supplementary material available at 10.1186/s12951-022-01623-2.

## Introduction

Triple-negative breast cancer (TNBC) is the most aggressive and dreadful subgroup of breast cancer, with the highest mortality rate and shortest median time of recurrence and death [1]. Clinically, the therapeutic regimen for TNBC treatment is quite limited due to its lack of response to hormonal therapies and HER-2 targeting therapies. Consequently, chemotherapy remains the mainstay [2]. Docetaxel (DTX) is a representative first-line drug for TNBC treatment, with many synergetic chemotherapy regimens studied for the heterogeneity of TNBC [3, 4]. Ginsenoside Rg3, the main active ingredient derived from Radix Ginseng, was approved as a commercial anti-cancer drug (Shenyi capsules) in 2004 in China and has been synergistically utilized with chemotherapy in the clinical treatment of breast cancers [5]. It was reported that Rg3 can improve the susceptibility of tumor cells to taxanes by inhibiting NF-κB signaling [6, 7]. Therefore, Rg3 is expected to enhance the cytotoxic effect of DTX as an adjuvant agent. However, it is difficult to realize the synergistic effect of DTX and Rg3 for the low bioavailability of Rg3 and the different in vivo fates between DTX and Rg3. Rg3 is easily degraded in the gastrointestinal tract and blood and cannot reach tumor site with DTX synchronously. In addition, nonionic surfactants are required due to the poor water-solubility of DTX and Rg3, which may induce serious adverse reactions, such as hypersensitivity reactions and peripheral neuropathy [8].

Given these drawbacks, surfactant-free nanocarriers have been well studied and developed as commercial drugs, such as Doxil® (liposomal formulation of doxorubicin) and Nanoxel-PM (docetaxel-loaded micelle). Among various nanocarriers, liposomes have been regarded as the most promising delivery system for the biocompatibility and the capability for co-delivery of combined drugs with different solubility [9]. Accordingly, approximately twenty liposomal products have passed into clinical use for cancer therapy [10]. However, nanotechnological chemotherapy has shown limited success in clinical translation [9]. Some studies demonstrated that despite the improved safety of free drugs, little benefit of Doxil® and Nanoxel-PM was observed for the overall survival of treated patients [8, 11]. It is mainly caused by two aspects: (1) inadequate tumor site-specific delivery and (2) tough tumor microenvironment (TME). It is said that liposomes can accumulate more at tumor site compared with free drugs by virtue of their enhanced permeability and retention (EPR) effect. However, perception about the potency of EPR effect in humans has been challenged in clinical practice. Vascular leakage in human tumors is not as significant as that in murine models, leading to the overestimation of the efficiency of EPR effect [9, 12]. Therefore, ligand-based active tumor-targeting strategies are steadily gaining attentions. Cancer cells tend to take up glucose at an elevated rate to meet their increased energy demands. The most widely expressed glucose transporter is glucose transporters 1 (Glut1), which is responsible for basal glucose uptake [13]. As a result, Glut1 is overexpressed and confers poor prognosis in a wide range of solid tumors in clinic such as TNBC, hepatic, pancreatic, esophageal, brain, renal, lung, cutaneous, colorectal, endometrial, ovarian and cervical cancers[14]. Therefore, Glut1 has been exploited as the clinically validated target for drug delivery in considerable tumor models. Glucose-modified liposomes have been designed to realize the active targeting to tumor cells via the interaction between glucose and Glut1 which is much more highly expressed on tumor cells than normal cells [13]. Although preclinical studies of ligand-modified liposomes for tumor therapy are compelling, none of them have been approved for clinical use [15]. The key challenge is that the surface modification of ligands or antibodies entails sophisticated synthesis and formulation procedures, posing challenges for large-scale production as well as the pharmacokinetics and toxicology evaluation [16]. That is the reason why most of the clinically approved nano-medicines have quite simplistic compositions. Therefore, a simple yet smart liposome is crucial in achieving the idea of “bench to bedside”.

Even if tumor targeting can be achieved, the efficacy of nanomedicines will still be limited by TME. Studies on anti-tumor strategies have been always centered on neutralizing tumor cells. However, the immunosuppressive TME and the physical penetration barrier created by the stromal cells, to a large extent, lead to poor responses to liposomal chemotherapy [17–19]. TNBC is a typical stroma-rich tumor [20] and is the most representative “cold” tumor with insufficient cytotoxic T lymphocyte infiltration [21]. Cancer-associated fibroblasts (CAFs) are the most predominant group among the interstitial cells and are critical modulators for the formation of dense extracellular matrix (ECM) and immunosuppressive TME [22, 23]. Researches on the depletion of CAFs are emerging to facilitate drug permeation and response and the sequential two-stage therapy was thereby applied, i.e., the first stage for CAFs exhaustion and the second stage for tumor cell neutralization [24, 25]. Although this strategy can theoretically improve the therapeutic effect, it tends to involve the ligands modification for tumor cells and CAFs targeting, and encapsulation of anti-cancer and anti-fibrotic agents, respectively. As mentioned above, no active tumor targeting liposomes have been approved in clinic for overcomplication, let alone the sequential therapy of two types of ligand-modified liposomes. Such two-step sequential dosing also makes it more difficult to develop clinical treatment regimens. Moreover, exhausting CAFs may abrogate crucial ECM components and promote tumor metastasis. Thus, it may be more feasible to inhibit the conversion and activation of CAFs instead of depleting them. Many studies have elucidated that smart tumor cells can promote the stroma-rich and immune-cold TME by secreting transforming growth factor beta (TGF-*β*) to educate CAFs formation and infiltration [22, 26]. Therefore, we speculated that suppressing TGF-*β* secretion from tumor cells to inactivate CAFs might reshape TME, enhance intratumor drug penetration, and achieve better therapeutic outcomes.

Surprisingly, in addition to improving the potency of chemotherapeutic drugs, Rg3 also possesses the anti-fibrotic and immunoregulatory capacities [27]. It has been reported that Rg3 is capable of blocking the tumor cells from TGF-*β* secretion, indicating its potential to hinder the induction role of tumor cells on CAFs precursors [28, 29]. Therefore, liposomes encapsulated with ginsenoside Rg3 and DTX can simultaneously realize TME remodeling and tumor cell neutralization by directly targeting tumor cells. The strategy can circumvent the hassle of the excessive complications associated with CAFs targeting requirements. Moreover, preliminary results from our laboratory showed that ginsenosides can act as a liposome membrane material instead of cholesterol, and, interestingly, ginsenoside liposomes also showed excellent tumor targeting properties [28–31]. Rg3 is an amphipathic material with hydrophilic glycosyl groups and a lipophilic steroidal structure similar to that of cholesterol (Additional file 1: Fig. S1). As a cholesterol analogue, it has the potential as a liposomal membrane stabilizer [29, 31]. Simultaneously, its glycosyls in hydrophilic part can theoretically stick out of the liposome surface, making it a perfect substrate for Glut1 overexpressed on tumor cells [32, 33]. Consequently, Rg3 can act as a liposomal membrane stabilizer, an adjuvant agent and an active tumor targeting ligand without additional chemical modifications.

Inspired by this deduction, a DTX-loaded Rg3 liposome (Rg3-Lp/DTX) was developed. In our hypothesis, Rg3 would prevent the formation and activation of CAFs by inhibiting the secretion of TGF-*β* from tumor cells and inhibiting the subsequent CAFs-induced physical and immune barriers in TME. As a result, Rg3-Lp/DTX would concentrate more and penetrate deeper into tumor to better exert synergistic cytotoxic effects. The strategy can eliminate the trouble of designing CAFs targeting nanocarriers, as well as the sequential two-stage administration. It can achieve tumor targeting, CAFs education, TME remodeling and enhanced cytotoxicity on tumor cells merely by replacing cholesterol with Rg3 without resorting to any complicated modifications and formulation processes. Therefore, the system has great clinical translation perspectives and can bridge the gap between laboratory trials and practical clinical applications.

## Results

### Characterizations of Rg3-Lp/DTX

**Scheme 1:**
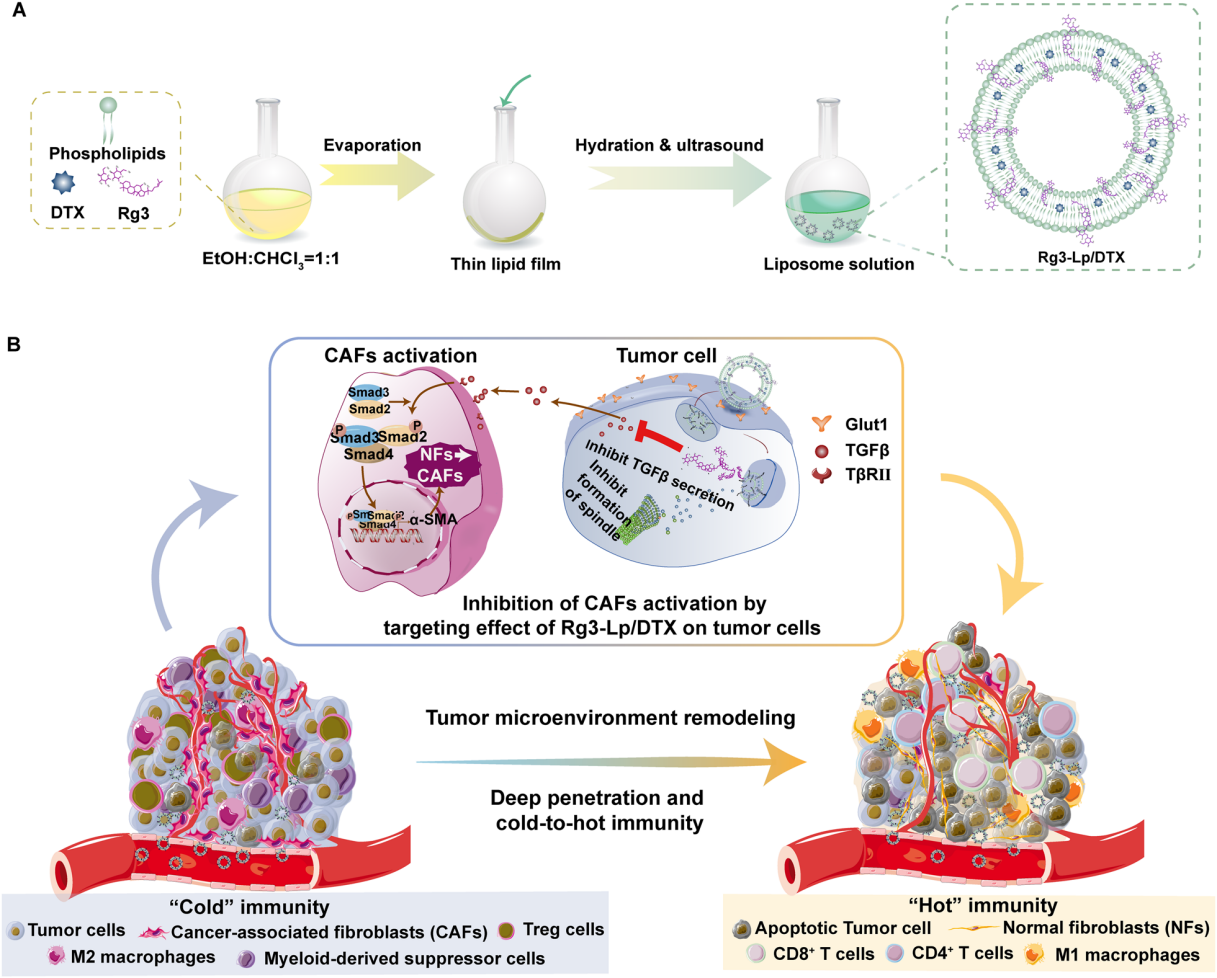
Schematic illustrations of the preparation of Rg3-Lp/DTX and its mechanism on TNBC inhibition. **A** Preparation process of Rg3-Lp/DTX. **B** The multiple functions of Rg3 as a tumor targeting material and crosstalk inhibitor between CAFs and tumor cells. Rg3-Lp/DTX can actively target to tumor cells through Glut1-Rg3 interaction. After uptake by tumor cells, Rg3 can prevent tumor cells from secreting TGF*β*, a tumor-secreted cytokine that educates the activation of CAFs. With the diminishment of activated CAFs, Rg3-Lp/DTX can penetrate deeper into tumor tissue to exert combined cytotoxic effect of DTX and Rg3 and convert the TEM from “cold” to “hot”. As a result, Rg3-Lp/DTX can achieve excellent anti-TNBC effect

**Fig. 1:**
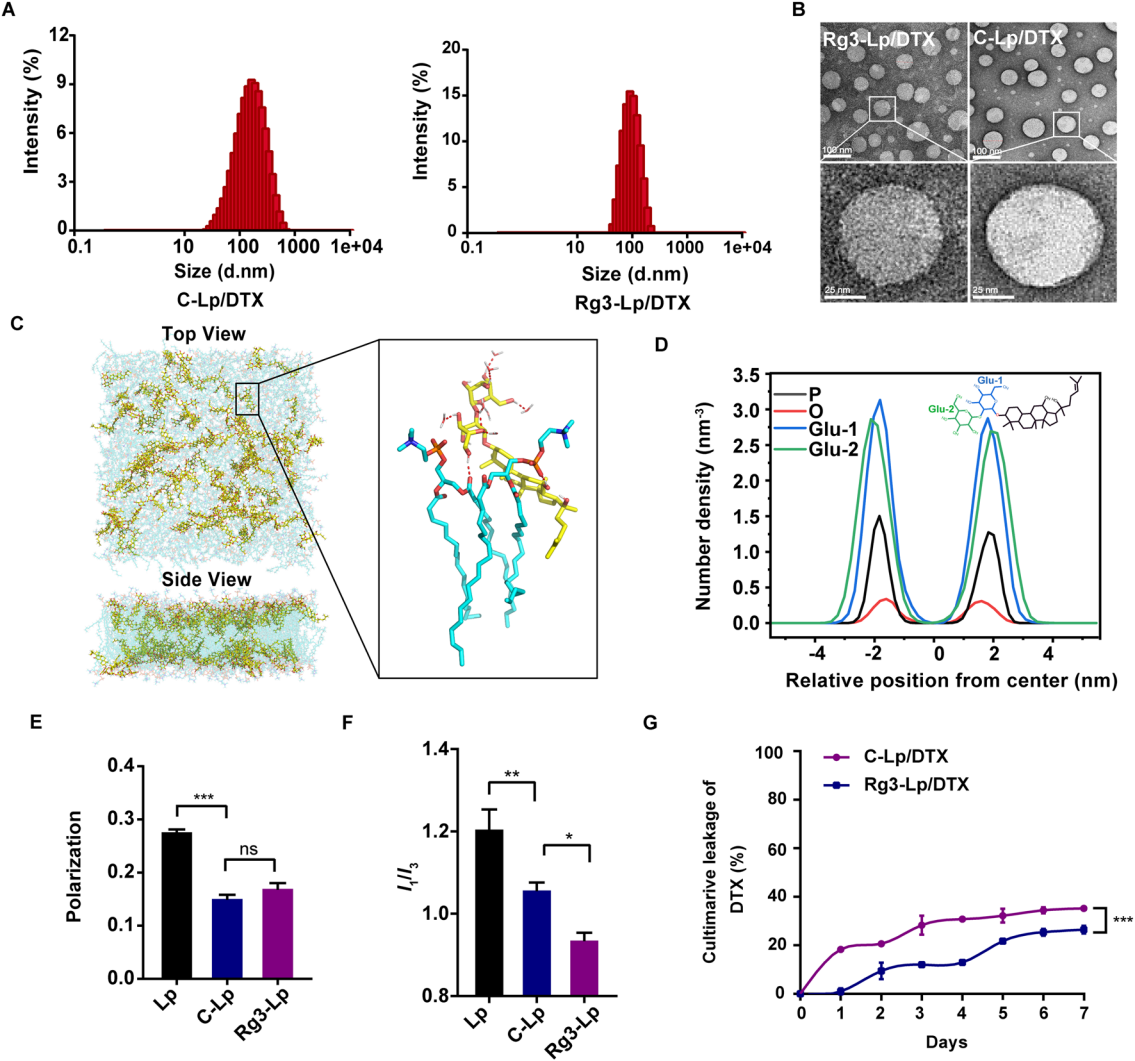
Characterization of the liposomes. **A** Size distribution of C-Lp/DTX and Rg3-Lp/DTX. **B** Transmission electron microscopy (TEM) images of C-Lp/DTX and Rg3-Lp/DTX. **C** Snapshot of the lipid bilayer of Rg3-Lp and typical co-ordinations of Rg3 with DSPC lipids and water molecules (H2O). (DSPC: blue sticks; Rg3: yellow sticks; H2O: O in red and H in white sticks; hydrogen-bonds: red dashed lines). **D** Density profiles of some major components of the membrane model. The phosphorus atom in DSPC, the oxygen atom connecting the glycosyl and skeleton, and the first glucose unit conjugated to the skeleton are colored in black, red, and blue, respectively. The second glucose unit in Rg3-Lp is colored in green. **E** Pyrene micro-polarity I1/I3 (378/383) in pure liposomes (Lp), C-Lp, and Rg3-Lp. **F** Fluorescence anisotropy of DPH obtained from Lp, C-Lp, and Rg3-Lp. **G** In vitro leakage stability of C-Lp/DTX and Rg3-Lp/DTX. *p < 0.05, **p < 0.01, and ***p < 0.001; Differences between two groups were analyzed with unpaired t-test, one-way ANOVA was performed to compare data among multiple groups; Error bars represent mean ± SD of three technical replicates

Rg3-Lp/DTX and C-Lp/DTX were prepared by thin-film hydration method (Scheme 1). DTX-loading efficiencies (LE) of Rg3-Lp/DTX and C-Lp/DTX were 7.1 ± 0.1% and 6.0 ± 0.1%, respectively (Table 1). The mean particle sizes of Rg3-Lp/DTX and C-Lp/DTX measured by dynamic light scattering (DLS) were 96.7 ± 4.5 nm and 136.8 ± 2.0 nm, respectively (Fig. 1A, Table 1). Transmission electron microscopy (TEM) images showed that both types of liposomes were spherical (Fig. 1B), indicating the successful construction of Rg3 liposomes. To locate Rg3 in the liposome membrane, molecular dynamics (MD) simulations on 1,2-dioctadecanoyl-sn-glycero-3-phosphocholine (DSPC)-Rg3 system of Rg3-Lp were conducted. The molecular arrangement of the stable Rg3-DSPC system obtained from MD simulation was shown in Fig. 1C. Rg3 was found to stably interact with the phospholipid molecules and interpenetrate in the lipid bilayer (Fig. 1C). From the typical conformation and interaction diagram of phospholipid molecules and Rg3 in Fig. 1C, it can be seen that the planar ring structure of Rg3 and the aliphatic chain at C17 can be embedded between the fatty acid tails of phospholipids, thus regulating the arrangement of phospholipid molecules in the bilayer. Simultaneously, the two glycosyl groups of Rg3 at C3 site, which are divided into endo-glycosyl (the first glycosyl unit conjugated to the skeleton, Glu-1) and exo-glycosyl (the second glucose unit, Glu-2), formed H-bond interactions with the polar heads of the phospholipid molecules and improve the stability of lipid membrane. To further reveal the precise position of the glycosyls of Rg3 in the bilayer, the density distribution of phosphorus atoms (P) in the polar head of phospholipid molecules, the oxygen atoms (O) connecting the planar ring and the glycosyl group of Rg3 and the Glu-1 and Glu-2 of Rg3 were analyzed. A symmetrical distribution of the headgroups of Rg3 and DSPC was exhibited in the density profiles along the bilayer’s center in Fig. 1D. The phosphorus atoms in the hydrophilic head of phosphorylcholine represented water–oil boundary between the hydrophobic and the aqueous regions [34]. The peak areas of Glu-1 and Glu-2 were broader than that of P. It suggested that some conformations of Rg3’s glycosyl moiety at C3-position went beyond the scope of DSPC and directly inserted into the water molecular layer (Fig. 1D). The phenomenon was also visually displayed in Fig. 1C, in which numbers of glycosyl units were exposed on the liposome membrane surface and penetrated deeply into the water phase, making it possible to actively interact with Glut1 overexpressed on tumor cells.

**Table 1:** Characterization of DTX-loaded liposomes

	Size (nm)	PDI	Zeta potential (mV)	EE (%)	LE (%)
C-Lp/DTX	136.8 ± 2.0	0.24 ± 0.03	− 31.2 ± 2.8	81.5 ± 1.3	6.0 ± 0.1
Rg3-Lp/DTX	96.9 ± 4.5	0.15 ± 0.02	− 27.8 ± 3.0	97.4 ± 1.3	7.1 ± 0.1

Error bars represent mean ± SD of three technical replicates

*EE* encapsulation efficacy, *LE* loading efficacy

As proved in Fig. 1C, D, Rg3 can spontaneously form a stable bilayer membrane structure with phospholipid molecules, indicating the potential of Rg3 as a liposome bilayer regulator. Therefore, to investigate the effect of Rg3 on the properties of lipid bilayer, membrane fluidity (Fig. 1E) and micro-polarity (Fig. 1F) of Rg3-Lp were investigated. The variation of membrane fluidity is related to the C17 side chain and plane ring structure of the regulators embedded between the tail of phospholipid, while the change of membrane micro-polarity is associated with the hydrogen bond interaction between the hydroxyl at C3 site of the regulator and the polar head of phospholipid [35]. As shown in Fig. 1E, the anisotropy of the membrane in Rg3-Lp was much smaller than that in pure phospholipid liposomes (Lp), and similar to that in C-Lp. The results indicated that Rg3 could increase the liposomal membrane fluidity similar to cholesterol. Due to the insertion of Rg3 or cholesterol molecules with the planar ring structure between phosphorylcholine molecules, the dispersion force between the fatty acids tails of phosphorylcholine molecules will be disrupted [36]. As a result, the lipid bilayer would be more fluid. Therefore, the membrane fluidity increased after the incorporation of Rg3 or cholesterol into the phospholipid bilayer (Fig. 1E). According to MD results, the side chain at C17 site and planar ring structure of Rg3 were interspersed between the fatty acid tails of phospholipids, which was mainly responsible for the regulation of membrane fluidity. Therefore, Rg3 showed a comparable effect on the fluidity of lipid membrane with that of C-Lp because of the similarity of them in the side chain and planar ring structures (Fig. 1E). In addition, to verify the interaction of the glycosyl units at C3 site of Rg3 with phospholipid molecules, micro-polarity of liposomes was measured (Fig. 1E). Pyrene is usually applied to measure the modulation of bilayer micro-polarity induced by Rg3 [35]. The fluorescence intensity ratio of pyrene *I1/I3* can reflect the polarity of the environment, which is related to the arrangement of acyl groups.^32^ A decrease in I1/I3 value implies a higher binding affinity between the membrane regulator and the phospholipid molecules, resulting in lower micro-polarity between the lipid bilayers. The tight connection is favorable for improving the membrane stability and encapsulation efficiency of hydrophobic drugs. The ratio of I1/I3 in Lp solution was 1.21 ± 0.05, while that in Rg3-Lp and C-Lp solution was 1.06 ± 0.02 and 0.94 ± 0.02, respectively (Fig. 1F). The results proved that the micro-polarity of Rg3-Lp was significantly lowered after the addition of Rg3, demonstrating that Rg3 formed intensive interactions with the polar head of phospholipids and further constituted hydrogen bond networks to stabilize the entire system [35]. Therefore, Rg3-Lp/DTX exhibited less leakage and better particle stability than C-Lp/DTX did during the storage period at 4℃ for 7 days in PBS (Fig. 1G and Additional file 1: Fig. S3). As shown in Fig. 1G, an obvious burst leakage of C-Lp/DTX (18.3%) was observed on day 1 versus the leakage of Rg3-Lp/DTX (1.0%), indicating that DTX leaked more easily from C-Lp/DTX than Rg3-Lp/DTX. As shown in Additional file 1: Fig. S3, the particle size and PDI of C-Lp/DTX increased significantly on day 4, whereas the size of Rg3-Lp/DTX remained stable, which may be due to the stronger interaction between Rg3’s glycosyl portion with phospholipid molecules.

### Enhanced cellular uptake of Rg3-Lp in tumor cells via Rg3-Glut1 interaction

**Fig. 2:**
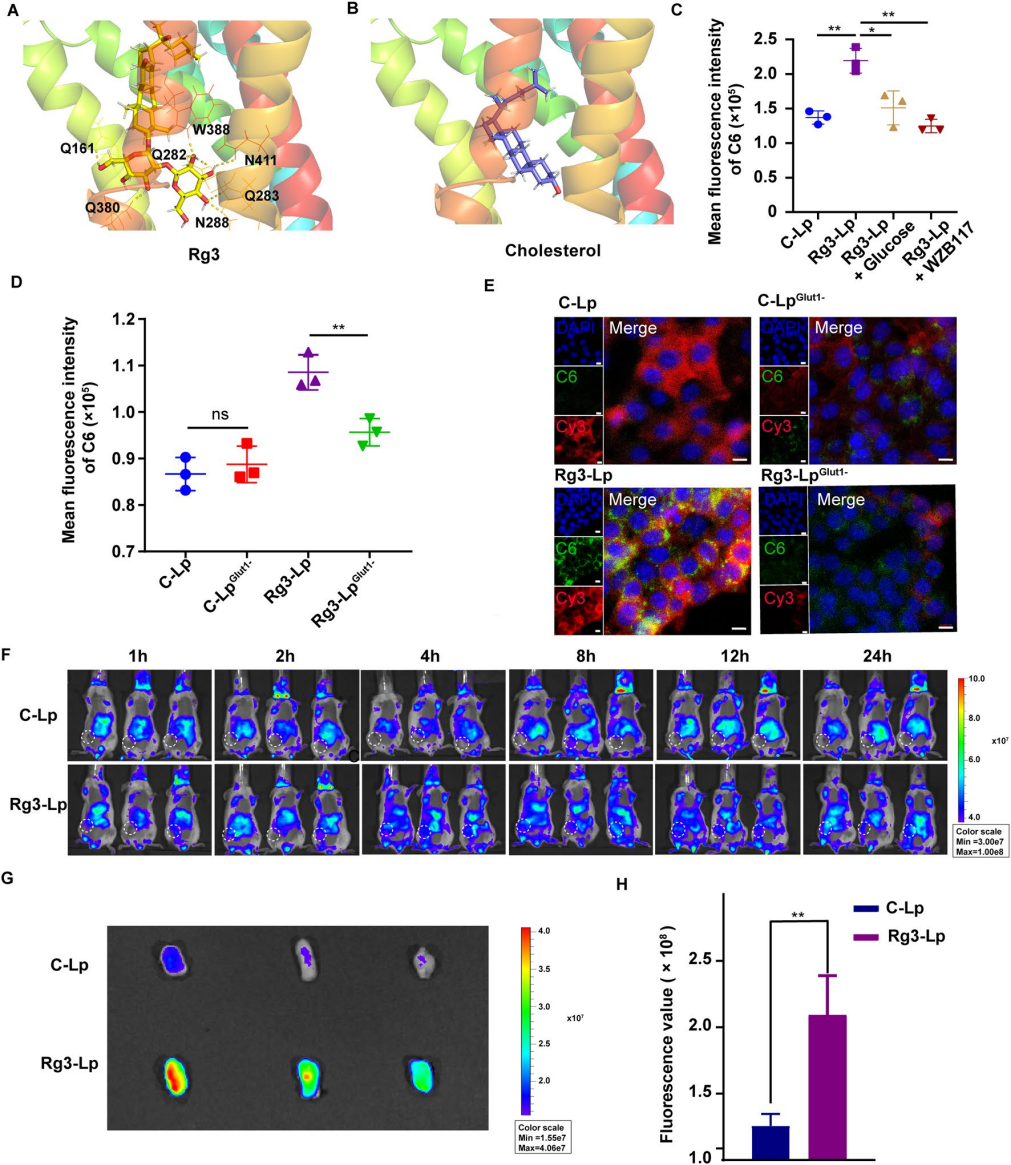
Tumor targeting ability of Rg3-Lp. Molecular docking of Glut1 with Rg3 (yellow sticks) (**A**) and cholesterol (blue sticks) (**B**), respectively. H-bond interactions between Rg3 and Glut1 were represented by the yellow dotted lines. **C** The quantitative analysis of cellular uptake of C-Lp/C6, Rg3-Lp/C6 and Rg3-Lp/C6 with Glut1 inhibitors in 4T1 cells via flow cytometry. The fluorescent intensity represents the mean fluorescence intensity (MFI) of C6-loaded liposome uptake by 4T1 cells. **D** Flow cytometry analysis of cellular uptake of C-Lp/C6 and Rg3-Lp/C6 in normal and 4T1^Glut1−^ cells, respectively. The fluorescent intensity represents the mean fluorescence intensity (MFI) of C6-loaded liposome uptake by 4T1 cells. **E** Representative confocal laser scanning microscope (CLSM) images of cellular uptake of C-Lp/C6 and Rg3-Lp/C6 in 4T1 cells before and after Glut1 knockdown. Blue: cell nucleus; green: liposomes; red: Glut1. Scale bar, 10 µm. **F** Biodistribution of the DID-labeled liposomes in 4T1 tumor-bearing mice at different time points after intravenous injection. **G** Ex vivo imaging of dissected tumors 24 h after injection of C-Lp/DiD and Rg3-Lp/DiD, respectively. **H** Semi-quantitative ROI values of mean fluorescence intensity at tumor sites. **p < 0.01; Data are shown as mean ± standard deviation of three technical replicates; Differences between two groups were analyzed with unpaired *t*-test, one-way ANOVA was performed to compare data among multiple groups

As illustrated in Fig. 1C, D, the glycosyls of Rg3 at the C3-position were oriented towards the water molecules and sticked out of the surface of the liposomal membrane, which could potentially interact with Glut1 overexpressed on tumor cells [37]. Therefore, molecular docking was carried out to explore the potential of Rg3 to interact with Glut1, respectively (Fig. 2A, B). It could be found that the glycosyl units of Rg3 was hydrogen-bonded to the surrounding polar residues of Glut1. The interacting residues were: W288, N288, N411, Q161, Q282, Q283, Q283 and Q380. Among these, Q282, Q283, W388, and N411 were crucial combination residues for ligand-Glut1 binding [38]. In addition, Q282 and Q283 were proved as key residues for glucose-Glut1 binding [39]. However, contrary to Rg3, cholesterol failed to be hydrogen-bonded to Glut1 because of the lack of a glycosyl moiety at its C-3 site (Fig. 2B). Therefore, it can be inferred that the glucosyls of Rg3 that exposed on the Rg3-Lp surface can interact with the corresponding amino acid residues in Glut1. At the same time, the cellular uptake of Rg3-Lp in TNBC tumor cells was investigated. The 4T1 cell/model was chosen because it robustly recapitulates many features of human TNBC [40], including the stroma-rich TME and Glut1 overexpression on tumor cells [41, 42]. The cellular uptake of Rg3-Lp in 4T1 cells was 1.7-fold higher than that of C-Lp and was significantly suppressed by WZB117, a specific Glut1 inhibitor [43], and glucose, a competitive Glut1 inhibitor (Fig. 2C and Additional file 1: Fig. S5). It suggested that Rg3 can actively target to tumor cells by interacting with Glut1 overexpressed in 4T1 cells.

The targeting mechanism was further verified by the cellular uptake assays of Rg3-Lp in 4T1 cells before and after Glut1 knockdown. As shown in Fig. 2D, the uptake of Rg3-Lp in Glut1-knockdown 4T1 cells (4T1^Glut−^) was significantly abated compared to that in normal 4T1 cells, whereas the uptake of C-Lp remained unchanged in 4T1^Glut−^ cells and normal 4T1 cells. After treatment with the liposomes, 4T1 cells were immunofluorescence (IF) stained for the visualization of Glut1 expression and were subjected to confocal laser scanning microscope (CLSM) imaging to observe Rg3-Lp—Glut1 binding. As shown in Fig. 2E, the yellow signals represented successful merging of Glut1 (red) signals and C6-loaded liposomes (green) signals. The fluorescence signal of Rg3-Lp was selectively localized at the sites revealing red signals and the merged signal of Rg3-Lp was much higher than that of C-Lp (Fig. 2E). Furthermore, the signal of Rg3-Lp in tumor cells was markedly diminished when the expression of Glut1 decreased (Fig. 2E). The results above synthetically demonstrated that Rg3 can be specifically taken up by tumor cells through the binding interaction between its glycosyl groups exposed on the liposomal surface and Glut1 overexpressed on tumor cells.

### Tumor tropism of Rg3-Lp

As proved in Fig. 2A–D, the glycosyls of Rg3 exposed on the liposomal surface could endow Rg3-Lp with the potential for active tumor targeting. The biodistribution of Rg3-Lp in tumor-bearing mice was monitored under in vivo imaging system (IVIS). DiD-loaded Rg3-Lp (Rg3-Lp/DiD) or DiD-loaded C-Lp (C-Lp/DiD) were intravenously injected into 4T1-bearing mice. Biodistribution of the DiD-loaded liposomes in 4T1-bearing mice were detected at different time points under IVIS (Fig. 2F). As shown in Fig. 2F, orthotopic tumor was located at the lower right quadrant of the abdomen of the mice marked with a white circle. The fluorescence signals could be observed at the breast tumor sites four hours after the administration of Rg3-Lp/DiD and showed stronger fluorescence signals from then on, while the signals at the tumor site of mice treated with C-Lp/DiD were hardly detected, indicating that Rg3-Lp accumulated more at tumor site than C-Lp. After 24 h, mice were sacrificed and the tumors and major organs were excised and imaged ex vivo under IVIS to observe the biodistribution of the liposome ex vivo (Fig. 2G, H and Additional file 1: Fig. S6). The targeting ability of Rg3 liposomes was demonstrated by imaging and semi-quantitative assays of the tumors excised from the mice at the ending point (Fig. 2G, H). The ROI value of the tumor injected with Rg3-Lp/DiD was almost two-fold higher of that from the tumor treated with C-Lp/DiD. It could be concluded that liposomes with Rg3 as membrane material can deliver drugs to tumor site more effectively and selectively than conventional cholesterol liposomes. One of the fundamental differences between malignant cancer cells and normal cells is that cancer cells obtain energy by an increased rate of aerobic glycolysis through the enhanced catabolism of glucose, instead of oxidative phosphorylation [13]. Cancer cells must elevate their glycolytic rate to meet the energy they need to proliferate rapidly and indefinitely. In order to achieve a glycolytic rate that is approximately 30-fold higher than that of normal cells, cancer cells must take up glucose at an elevated rate [13]. The glycolysis rate strongly depends on the upregulated expression and activity of Glut1, with a 10–12-fold higher expression in tumor cells than that in normal cells [14, 44]. As a result, overexpression of Glut1 has been recognized as one of the hallmarks of cancer cells. Therefore, Rg3-Lp can accumulate more at tumor sites than C-Lp via the interaction between the glucose moiety of Rg3 and Glut1 overexpressed on tumor cells.

### Enhanced cytotoxicity of Rg3-Lp/DTX against tumor cells

**Fig. 3:**
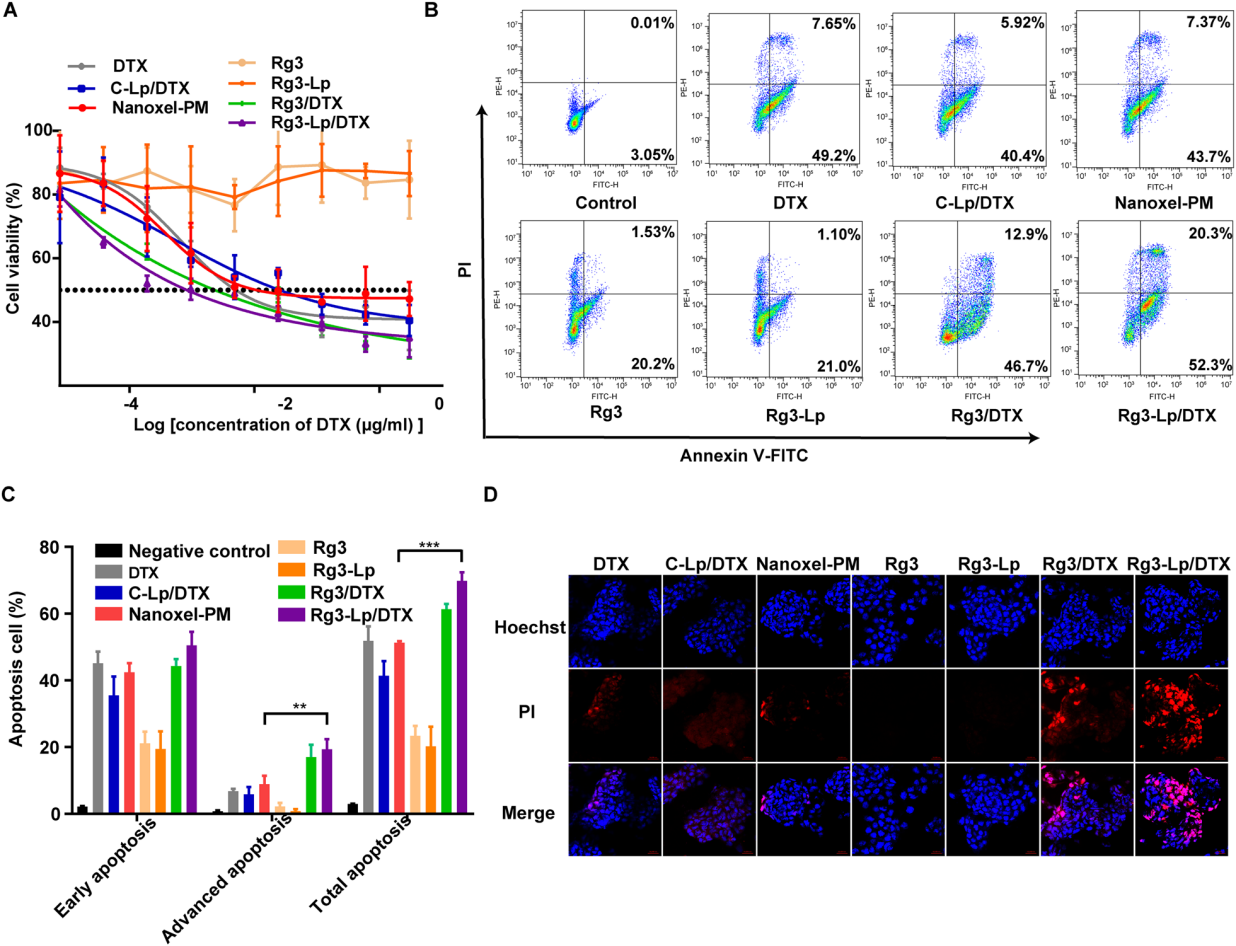
In vitro cytotoxicity effect of Rg3-Lp. **A** MTT assay of DTX, Rg3, Rg3-Lp and different DTX formulations against 4T1 cells. (n = 6) **B** Flow cytometry detection of cell apoptosis in 4T1 cells incubated for 48 h with DTX, C-Lp/DTX, Nanoxel-PM, Rg3, Rg3-Lp, Rg3/DTX and Rg3-Lp/DTX, respectively. Quantitative (**C**) and qualitative (**D**) cell apoptosis of PBS (negative control), DTX, C-Lp/DTX, Nanoxel-PM, Rg3, Rg3-Lp, Rg3/DTX and Rg3-Lp/DTX on 4T1 cells. (n = 3) Blue signal: Hoechst; Red signal: propidium iodide (PI). ***p < 0.001; Differences between two groups were analyzed with unpaired t-test, one-way ANOVA was performed to compare data among multiple groups

The in vitro cytotoxicity of DTX and different DTX-loaded liposomes on 4T1 cells was measured by MTT assays (Table 2, Fig. 3A). Unlike C-Lp/DTX (IC_50_ = 25.4 ng/ml) and Nanoxel-PM (IC_50_ = 10.9 ng/ml), Rg3-Lp/DTX showed the strongest cytotoxicity effect (IC_50_ = 0.8 ng/ml) (Table 2, Fig. 3A). To explain this phenomenon, IC_50_ value between DTX and simple Rg3 and DTX mixture (Rg3/DTX) group were compared and we found that the IC_50_ value of Rg3/DTX group was half that of DTX group even though the cytotoxic effect of Rg3 or Rg3-Lp were obviously weaker than that of DTX (Table 2, Fig. 3A). It indicated that Rg3 was an adjuvant drug for DTX which can enhance the cytotoxicity of DTX, but showed much lower cytotoxic effect compared to DTX when administered alone. It has been reported that Rg3 is capable of sensitizing tumor cells to chemotherapeutic drugs, and could enhance the inhibitory effects of docetaxel on cancer cells while its own cytotoxicity was not significant as chemotherapeutic drugs [45]. Moreover, the IC_50_ value of Rg3-Lp/DTX group was about 0.35 times that of Rg3/DTX group (Table 2, Fig. 3A). It might be ascribed to the glycosyl chains of ginsenoside Rg3, through which Rg3-Lp can enhance cellular uptake mediated by the specific binding between Glut1 and Rg3 as proved above and accordingly deliver more DTX and Rg3 into tumor cells. Therefore, the strong 4T1 cytotoxic effect of Rg-Lp/DTX was the combined result of its tumor-targeting ability and its synergistic effect with DTX.

**Table 2:** IC_50_ value of DTX and different DTX-loaded carriers

Group	IC_50_ value (ng/ml)
DTX	5.1
C-Lp/DTX	25.4
Nanoxel-PM	10.9
Rg3	N/A
Rg3-Lp	N/A
Rg3/DTX	2.3
Rg3-Lp/DTX	0.8

Cell apoptosis assay was further conducted to verify the cytotoxicity of Rg3-Lp/DTX. As illustrated in Fig. 3B, C, the results of cellular apoptosis experiment were similar to those of MTT assays. Rg3-Lp alone showed significantly weaker pro-apoptosis effect than DTX. However, encapsulation of DTX in Rg3 liposomes can significantly facilitate late apoptosis and enhance total apoptosis rate. The notable enhanced apoptosis effect of Rg3-Lp/DTX was further verified by qualitative observation under an inverted fluorescence microscope (Fig. 3D). The effect mainly relied on the facilitated cellular uptake of Rg3-Lp/DTX and chemo-sensitization effect of Rg3.

### Inhibition of tumor growth by Rg3-Lp/DTX

**Fig. 4:**
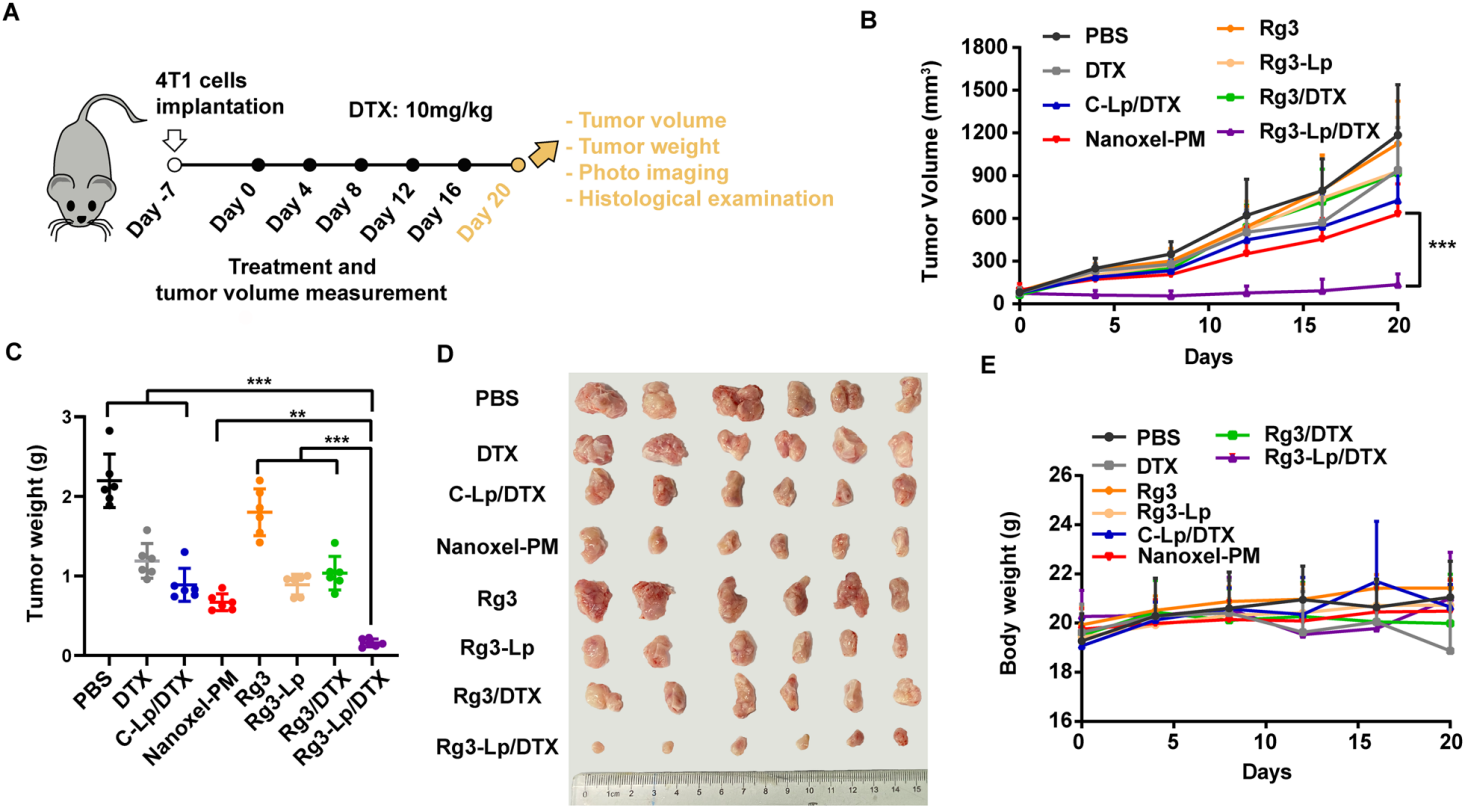
Rg3-Lp/DTX inhibited tumor growth in the 4T1 orthotopic mouse model. **A** Experimental scheme of the treatment schedule for orthotopic TNBC therapy. **B** Tumor growth curves of 4T1-bearing mice treated with PBS, Rg3, Rg3-Lp and different DTX formulations, respectively. n = 6 in each group. One-way ANOVA was performed to compare the tumor volumes among multiple groups at the endpoint. **C** Tumor weight of 4T1-bearing mice at the ending point of the treatment. (n = 6) (**D**) The photo of tumors excised from 4T1-bearing mice at day 20. (n = 6) (**E**) The body weight curve of 4T1-bearing mice treated with PBS, Rg3, Rg3-Lp and different DTX formulations, respectively. (n = 6) **p < 0.01, and ***p < 0.001; One-way ANOVA was performed to compare data among multiple groups

In vivo antitumor efficacy of the drugs was evaluated in tumor-bearing mice after treatment with PBS, DTX, C-Lp/DTX, Nanoxel-PM, Rg3, Rg3-Lp, Rg3/DTX and Rg3-Lp/DTX respectively once every four days via caudal vein. Tumor volume and body weight of each mouse were monitored at the same time of administration and none of the tumor-bearing mice after different treatment dead during the monitoring period. At day 20, tumors of each group were dissected, weighted and photographed (Fig. 4A). As shown in Fig. 4B, the growth of tumors treated with C-Lp/DTX and Nanoxel-PM was slightly slower than that of free DTX group. However, after replacing cholesterol with Rg3, the growth of tumors treated with Rg3-Lp/DTX was almost arrested. The results showed that Rg3-Lp/DTX significantly diminished the tumor volume and weight (Fig. 4C, D). In addition, tumor volume and weight of Rg3 group were almost the same as those of PBS group, but the tumor growth was significantly inhibited when Rg3 was prepared into Rg3-Lp (Fig. 4C, D), which is comparable to the effect of C-Lp/DTX group. Since Rg3 itself did not exert cytotoxicity effect as proved above, it suggests that Rg3 may act via other pathways to regulate tumor growth. The results in Fig. 4E indicates that Rg3-Lp/DTX presented significantly improved antitumor activity with no loss of body weight, whereas the body weight decreased slightly after the treatment with free DTX, which may be attributed to its systemic toxicity.

### Rg3 inhibited the activation of CAFs based on TGF-*β*/Smad pathway

TGF-*β*/Smad pathway plays an essential role in the conversion of normal fibroblasts (NFs) to cancer-associated fibroblasts (CAFs) [41]. TGF-*β* secreted by tumor cells binds to and activates receptors on the precursors of CAFs, resulting in the phosphorylation of Smad2 and Smad3. Then, complexes between phosphorylated Smad2/3 and Smad4 are formed and translocated into the nucleus to bind with the associated DNA strands and disrupt their transcription, thereby undermining the activation of fibroblasts (cancer associated fibroblasts, namely CAF) in TME, which typically involves the up-regulation of markers such as α-SMA [25, 46]. CAFs along with the secreted dense extracellular matrix (ECM) form a stiff physical barrier that inhibits the penetration of liposomes.

**Fig. 5:**
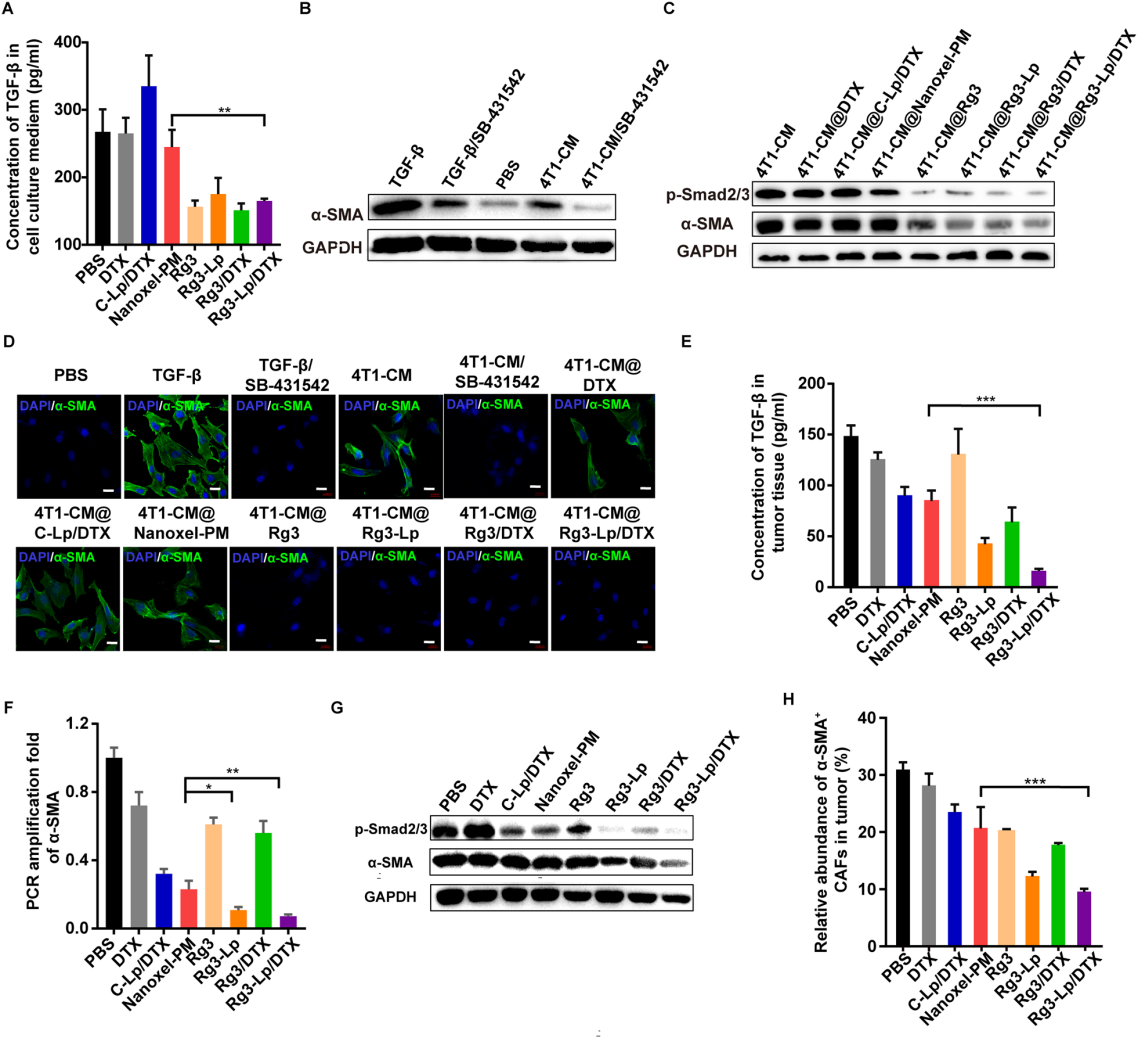
Inhibition effect of Rg3 on CAFs formation and activation. **A** Concentration of TGF-*β* in 4T1-cultured medium (CM) after the treatment of PBS, DTX, C-Lp/DTX, Nanoxel-PM, Rg3, Rg3-Lp, Rg3/DTX and Rg3-Lp/DTX, respectively. (n = 3) **B** Western blot detection of α-SMA and GADPH on 3T3 cells after treated with TGF-*β* (20 ng/ml), TGF-*β*/SB-431542, PBS, different conditioned 4T1 medium and 4T1-CM/SB-431542. **C** Western blot detection of α-SMA, p-Smad2/3 and GADPH on 3T3 cells treated with different conditioned 4T1 medium. **D** IF observation over α-SMA in 3T3 cells after treatment with TGF-*β* (20 ng/ml), TGF-*β*/SB-431542, PBS, 4T1-CM, 4T1-CM /SB-431542 and different conditioned 4T1 medium. **E** ELISA assay of the concentration of TGF-*β* in tumor tissues after the treatment of PBS, DTX, C-Lp/DTX, Nanoxel-PM, Rg3, Rg3-Lp, Rg3/DTX and Rg3-Lp/DTX, respectively. (n = 3) **F** q-PCR assay of α-SMA level in tumor tissues after the treatment of PBS, DTX, C-Lp/DTX, Nanoxel-PM, Rg3, Rg3-Lp, Rg3/DTX and Rg3-Lp/DTX, respectively. (n = 3) **G** Western blot detection of p-Smad2/3, α-SMA, *β*-actin and GADPH in tumors after treatment with PBS, DTX, C-Lp/DTX, Nanoxel-PM, Rg3, Rg3-Lp, Rg3/DTX and Rg3-Lp/DTX, respectively. **H** Flow cytometry analysis of activated CAFs in tumor after treatment with PBS, DTX, C-Lp/DTX, Nanoxel-PM, Rg3, Rg3-Lp, Rg3/DTX and Rg3-Lp/DTX, respectively. (n = 3) *p < 0.05, **p < 0.01, and ***p < 0.001; Differences between two groups were analyzed with unpaired t-test, one-way ANOVA was performed to compare data among multiple groups; Error bars represent mean ± SD of three independent experiments

As cancer cell-derived TGF-*β* is a prominent CAFs-inducer, TGF-*β* secretion in 4T1 conditioned medium (CM) was measured by ELISA assay after different treatments. As shown in Fig. 5A, the concentration of TGF-*β* in 4T1 cultured medium was significantly decreased after Rg3 treatment. The TGF-*β* concentration in Rg3-Lp/DTX group was only half of that in C-Lp/DTX group. NFs are the predominant precursors of CAFs [41]. TGF-*β* secreted form tumor cells can transform the normal paraneoplastic fibroblasts into CAFs gradually during tumor progression [45]. Mouse embryonic fibroblast 3T3 is a representative murine normal fibroblast which is sensitive to the cytokine stimulation [47]. Artificial CAFs are usually made in vitro by activating 3T3 cells using TGF-*β* or CM from tumor cells [47–49]. Therefore, to simulate education role of tumor cells on normal fibroblasts-to-CAFs transformation, normal fibroblast 3T3 cells was cultured with tumor CM after different treatment. By characterizing phenotypic changes in 3T3 cell line, the effect of Rg3 on the suppression of tumor-induced CAFs activation can be evaluated. To investigate the phenotypic changes of CAFs precursors to TGF-*β*, we measured α-SMA expression, a representative CAFs marker, of 3T3 cells after different treatments. The level of α-SMA expression of 3T3 cells was obviously enhanced after stimulation with TGF-*β* (20 ng/ml) and 4T1-CM, respectively, when compared with that of PBS group (Fig. 5B). Furthermore, with the addition of SB-431542, a TGF-*β*/Smad inhibitor, the expression of α-SMA in 3T3 cells was lowered, indicating that TGF-*β* was a dominant factor in 4T1-CM that led to CAFs activation (Fig. 5B). 3T3 cells were then cultured with different 4T1-CM which were harvested from the cultured medium of 4T1 cells after treatment with PBS, DTX, C-Lp/DTX, Nanoxel-PM, Rg3, Rg3-Lp, Rg3/DTX or Rg3-Lp/DTX. The expression levels of p-Smad2/3 and α-SMA were shown in Fig. 5C and Additional file 1: Fig. S9, from which we can found that high level of p-Smad2/3 and α-SMA was detected in 3T3 cells when cultured with 4T1-CM which was extracted from 4T1 conditioned medium pretreated with PBS (4T1-CM), DTX (4T1-CM@DTX), C-Lp/DTX (4T1-CM@C-Lp/DTX) and Nanoxel-PM (4T1-CM@Nanoxel-PM), respectively. On the contrary, significantly lowered expression of p-Smad2/3 and α-SMA expression was observed in 3T3 cells when cultured with 4T1-CM which was extracted from 4T1 cultured medium pretreated with all Rg3 containing group, including Rg3 (4T1-CM@Rg3), Rg3-Lp (4T1-CM@Rg3-Lp), Rg3/DTX (4T1-CM@Rg3/DTX) and Rg3-Lp/DTX (4T1-CM@ Rg3-Lp/DTX) (Fig. 5C and Additional file 1: Fig. S9). The results demonstrated the outstanding effect of Rg3 on decreasing the expression of p-Smad2/3 and α-SMA. The tendency was consistent with immunofluorescence (IF) staining results of α-SMA expression in Fig. 5D. The green signal which represents the α-SMA expression in 3T3 cells was conspicuous when treated with TGF-*β*, 4T1-CM and 4T1-CM@DTX, respectively, while the signal remained quite weak after the treatment with 4T1-CM@Rg3 or additional SB-431542 (Fig. 5D). Therefore, it can be speculated that Rg3 can inhibit the interaction between tumor cells and CAFs by downregulating TGF-*β* secretion of tumor cells and the subsequent TGF-*β*/Smad signaling of CAFs.

To verify the deduction, in vivo analysis of the corresponding indicators was carried out. The level of TGF-*β* in tumor tissues of different groups was measured by ELISA kit and the gene expression of α-SMA was analyzed by q-PCR assays. As revealed in Fig. 5E, F, the interaction between tumor cells and CAFs led to hyperactivation of TGF-*β* pathway, involving a-SMA induction and myofibroblast trans-differentiation. The neoplastic TGF-*β* concentration in Rg3-Lp and Rg3-Lp/DTX group was nearly half of that in C-Lp/DTX group and consequently, the a-SMA gene expression of tumor tissues in Rg3-Lp and Rg3-Lp/DTX group was decreased to one-third of that in C-Lp/DTX group (Fig. 5E, F). Moreover, the protein expression p-Smad2/3 and a-SMA of tumor tissue in Rg3-Lp and Rg3-Lp/DTX group was obviously lower than that in C-Lp/DTX group, indicating that Rg3 can effectively inhibit the conversion to CAFs via tumor TGF-*β* secretion and TGF-*β*/Smad signaling suppression (Fig. 5G and Additional file 1: Fig. S10). As a result, the abundance of activated CAFs was significantly reduced in tumor tissue by Rg3-Lp and Rg3-Lp/DTX (Fig. 5H). Unlike the in vitro results showing that Rg3 and Rg3-Lp have comparable inhibition efficacy of tumor-CAFs interaction, the level of TGF-*β* concentration and signaling in tumors of Rg3-Lp group was much lower than that in free Rg3 group (Fig. 5E–H) in vivo owing to the enhanced targeting delivery capacity when Rg3 was formulated into liposomes.

### Enhanced tumor penetration capacity of Rg3-Lp

**Fig. 6:**
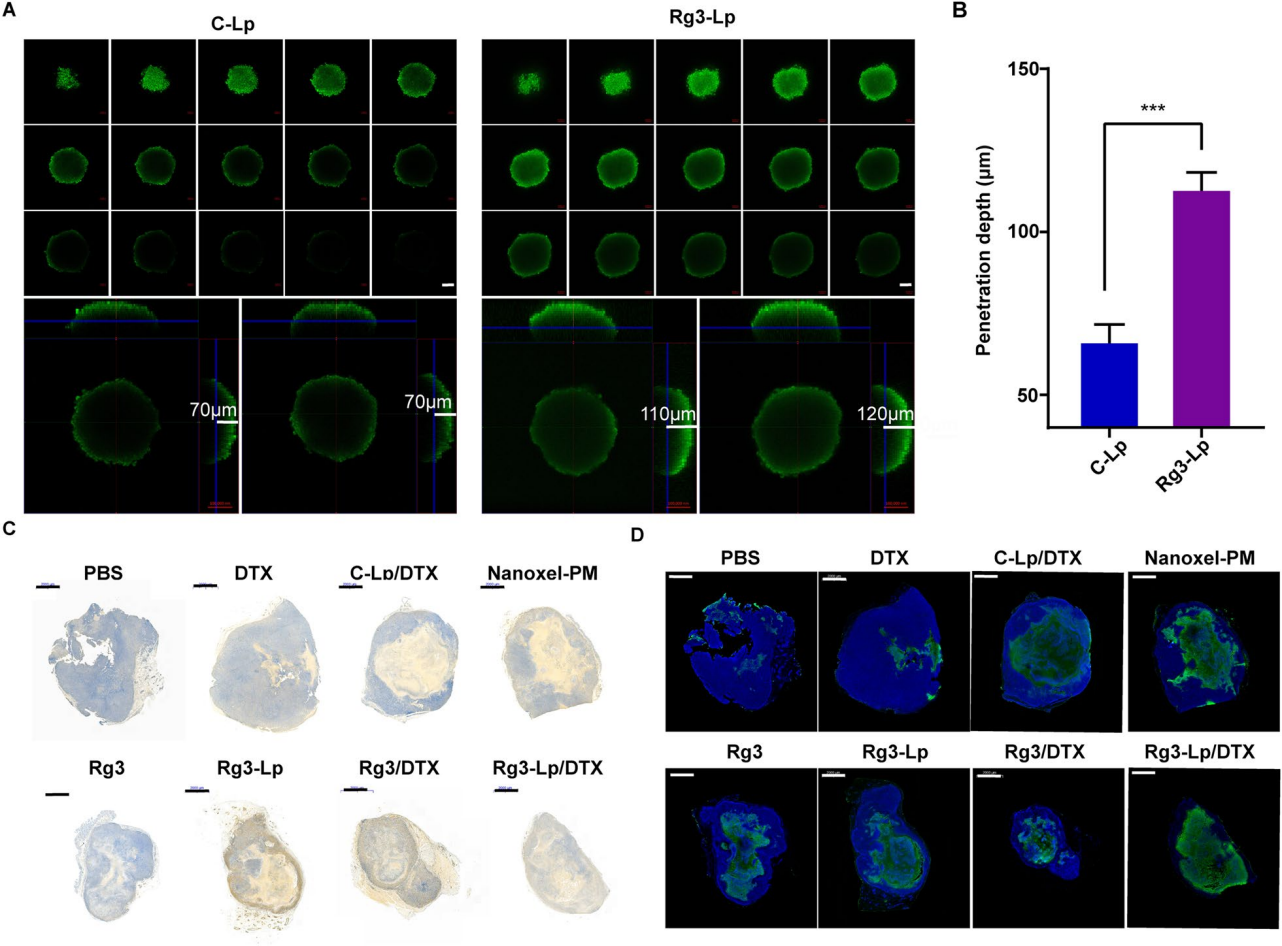
Enhanced tumor penetration ability of Rg3-Lp. (**A**) Fluorescence analysis of the 3D 4T1/3T3 tumoral spheroids accumulation and penetration of C-Lp/C6 and Rg3-Lp/C6 by confocal microscopy imaging. scale bar: 100 μm. (**B**) Penetration depth of different C6-loaded liposomes into 4T1/3T3 spheroid. (n = 3). Qualitative analysis of the signal of CAFs (**C**) identified by α-SMA antibody staining (blue) and (**D**) apoptotic tumor cells identified by TUNEL staining (green) in tumor section after different treatment. Scale bar: 2 mm. ***p < 0.001; Unpaired t-test was used for analysis of differences between two groups. Error bars represent mean ± SD of three independent experiments

An obvious decrease in CAFs abundance in tumors could be observed in Rg3-Lp and Rg3-Lp/DTX groups compared with that in PBS group and other DTX-loaded nanocarriers, which allowed Rg3-based liposomes to access tumor cells without obstacles raised from CAFs (Fig. 5G, H). As shown in Fig. 6A, Rg3-Lp could penetrate more deeply into 3D stroma-rich tumor spheroids composed of 4T1 tumor cells and 3T3 cells, and stronger fluorescence intensity and deeper penetration distance could be observed in tumor spheroids treated with Rg3-Lp/C6. Moreover, the depth of penetration of Rg3-Lp and C-Lp in tumor sphere was measured to be 66.7 ± 5.8 μm and 113.3 ± 5.8 μm (Fig. 6B), respectively, implying that Rg3 could significantly improve the tumor penetration ability of the liposomes. To analyze the association between the level of CAFs and penetration depth of the liposomes, tumor sections from each group were immunohistochemically stained and fully scanned for the visualization of α-SMA and TUNNEL signals (Fig. 6C, D). The TUNNEL signals in the fully scanned images of tumor slices can reflect the penetration depth in the tumor, since only when the liposomes reach the site can their cytotoxic effect work on the cells. Surprisingly, the sites with positive tunnel signals were found to basically coincide with those with weak α-SMA signals (Fig. 6C, D), which means that the depth of tumor penetration was negatively correlated with the abundance of activated CAFs. As shown in Additional file 1: Fig. S14, the significant decrease in collagen deposition could be observed in Rg3-Lp and Rg3-Lp/DTX group compared with that in PBS group and other DTX formulations, which allowed the liposomes to reach tumor cells without obstacles raised from CAFs and their secreted ECM. Therefore. as shown in Fig. 6C, D, the tumor slices in Rg3-Lp/DTX group showed the strongest tunnel signal and the widest tunnel signal area, suggesting the excellent tumor penetration ability of the system.

### Activated tumor immune microenvironment by Rg3-Lp/DTX

**Fig. 7:**
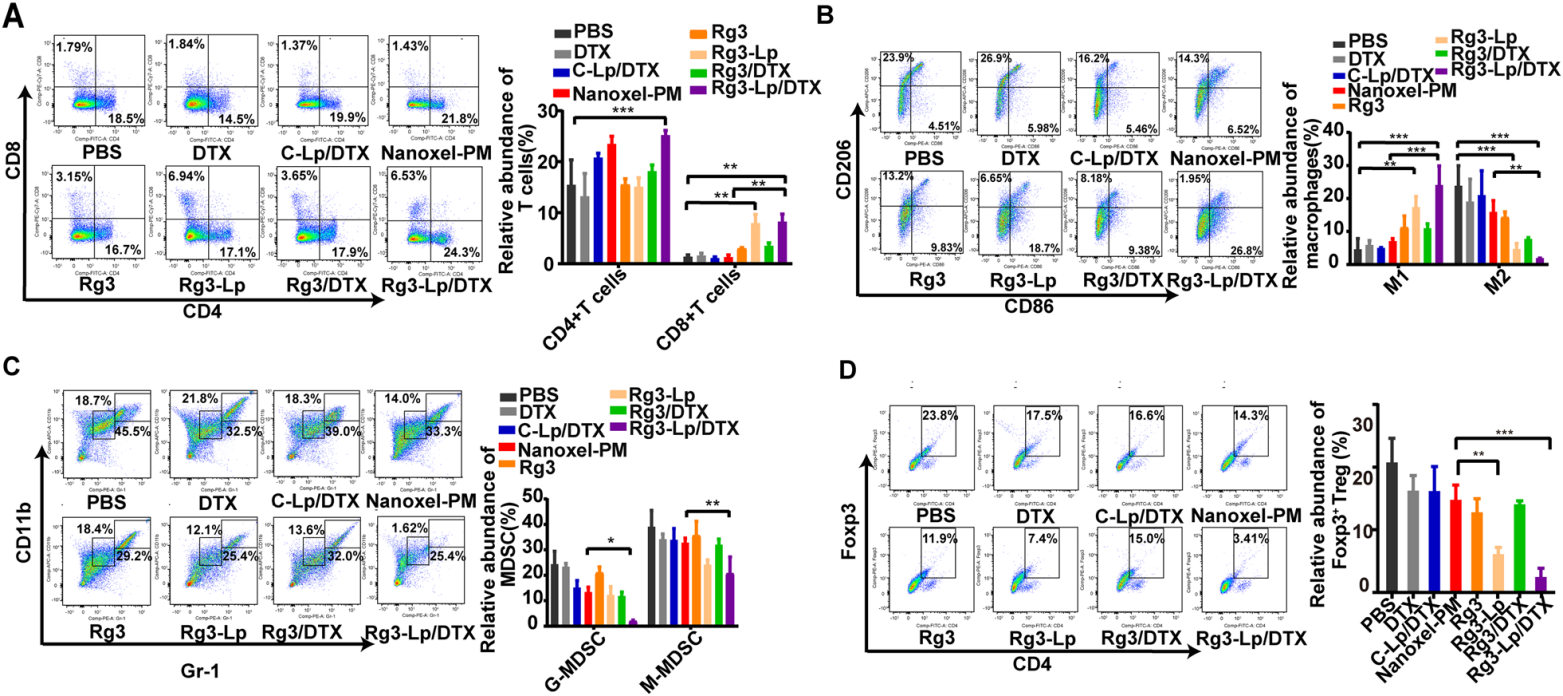
Analysis of immune cells in TME. **A** Flow cytometric and histogram analysis of the relative abundance of CD4^+^ or CD8^+^ T cells over total lymphocyte cells (CD45^+^ cells) in tumors treated with PBS, DTX, C-Lp/DTX, Nanoxel-PM, Rg3, Rg3-Lp, Rg3/DTX and Rg3-Lp/DTX, respectively. **B** The relative abundance of CD86 positive M1-type and CD206 positive M2-type macrophages over total macrophages populations (CD45^+^F4/80^+^) in tumors treated with different groups. **C** Flow cytometric and histogram analysis of the relative abundance of CD11b^+^/Gr-1^+^ MDSC cells over total lymphocyte cells (CD45^+^ cells) in tumors treated with different groups, including Gr-1^high^CD11b^+^ granulocytic (G-MDSC) and Gr-1^int^CD11b^+^ monocytic (M-MDSC) MDSC. **D** Flow cytometry gating and histogram analysis of the relative abundance of CD4^+^FoxP3^+^ Treg cells over total lymphocyte cells (CD45^+^ cells) in tumors. *P < 0.05, **P < 0.01, and ***P < 0.001; One-way ANOVA was performed to compare data among multiple groups; Error bars represent mean ± SD of three independent experiments

Breast cancer is categorized as a cold tumor, in which effector T cells are either excluded from the tumor area or taken away from being in contact with tumor cells. Apart from acting as a barrier for the penetration of drugs into the tumor area, the dense stroma like CAFs presumably creates an immunosuppressive tumor microenvironment, including low cytotoxic T cells infiltration, M1 to M2 polarization and enrichment of immunosuppressive cells [23]. Therefore, tumor-infiltrating lymphocytes were quantified by flow cytometry to examine whether Rg3-Lp/DTX could turn the tumor immunity from cold into hot by attenuating the activation of CAFs. The immunostimulatory effects of Rg3-Lp/DTX were shown in Fig. 7. A notable increase in CD4^+^ and CD8^+^ T cells in the tumors treated with Rg3-Lp/DTX could be observed, indicating that tumor immunity was getting hotter (Fig. 7A). As depicted in Fig. 7B, the number of M2 tumor-facilitating macrophages was significantly reduced in Rg3-Lp/DTX group. In contrast, the number of M1 tumor-suppressing macrophages was dramatically increased by Rg3-Lp/DTX, which might be attributed to the inhibition of TGF-*β* signaling and CAFs activation induced by Rg3-Lp. It has been reported that CAFs are important inducers for the transformation from M1- to M2- macrophages [23]. As proved in Fig. 5 3T3 cells were activated into CAFs after incubating with 4T1-CM since it contained high levels of TGF-*β* secreted by tumor cells. When treated with CAFs cultured medium that was collected from 3T3 cells pretreated with 4T1-CM (3T3-CM@4T1-CM), M1-phenotype macrophages were differentiated into M2-phenotype, indicating the induction role of CAFs on M1 to M2 differentiation (Additional file 1: Fig. S13). It has been proved that Rg3 could significantly decrease the level of activated CAFs by inhibiting tumor secretion of TGF-*β* (Fig. 5A–H). As a result, when incubated with 3T3 cultured medium that was collected from 3T3 cells pretreated with 4T1-CM@Rg3-Lp (3T3-CM@(4T1-CM@Rg3-Lp)), the relative abundance of M2 macrophages converted from M1 macrophages was significantly reduced than that in 3T3-CM@4T1-CM group (Additional file 1: Fig. S13). Therefore, Rg3-Lp is potential to inhibit the M1 to M2 shift induced by CAFs and raise the M1/M2 ration in TME via the suppression of tumor-induced CAFs activation. In addition, the number of immunosuppressive regulatory T cells (Treg) and myeloid-derived suppressor cells (MDSC) was obviously decreased in Rg3-Lp/DTX treated groups (Fig. 7C, D). In summary, Rg3-Lp/DTX created a more immune-active microenvironment compared to routine C-Lp/DTX and Nanoxel-PM with more CD4^+^ and CD8^+^ T cells, decreased MDSCs and Tregs and increased M1/M2 ratios, mainly due to the targeting delivery of Rg3 and its effect on the inhibition of CAFs, a cold immunity inducer [50].

## Discussion

Synergetic chemotherapy regimens have been widely applied in clinic for the high molecular heterogeneity of TNBC [3, 4] Ginsenoside Rg3 has been applied in synergy with chemotherapeutic agents in breast cancer therapy to optimize the clinical outcomes of antitumor drugs.^5^ We found that the cytocidal and pro-apoptotic effect of DTX was significantly improved when administered in combination with Rg3 even though Rg3 showed much lower cytotoxicity to 4T1 cells compared to DTX. The IC_50_ value of Rg3/DTX was 0.45 times that of DTX and the percentage of late apoptotic cells in Rg3/DTX group was nearly twice that in DTX group. Therefore, Rg3 is potential as an adjuvant drug to enhance the efficacy of chemotherapy for TNBC treatment.

Despite the enhanced tumor cytotoxic effect of the combination of Rg3 and DTX in vitro, the co-administration regimen is far from satisfactory in vivo caused by the low bioavailability of Rg3 and different systemic distribution between Rg3 and DTX [3, 4]. Thus, liposomes have been extensively investigated as effective carriers for co-delivery of combined drugs [51]. Due to the controversy regarding the difference of EPR effect between human and experimental animal models, researchers are increasingly focusing on ligand-modification active targeting to realize tumor site-specific aggregation of the loaded agents. Unfortunately, no active targeting nanocarriers have been approved for clinical use to date for the complex ligand chemical modification process and the excessively laborious formulation process that sacrifices the clinical translational feasibility.

In our study, we found that Rg3 could help liposomes achieve tumor active targeting without resorting to any complex preparations. We found that the structure of Rg3 satisfies the requirements of a liposomal membrane regulator to interact with phospholipid molecules and stabilize the liposome bilayer [52]. Therefore, cholesterol was replaced with Rg3 to construct Rg3-Lp/DTX. Rg3-Lp/DTX had the similar morphology and drug loading efficacy to C-Lp/DTX, indicating that Rg3 was successfully utilized as a liposome membrane regulator. MD simulation of Rg3-phospholipids system proved that Rg3 can stably intercalate into the lipid bilayer and form intensive hydrogen bonds with phospholipid molecules through its hydroxyl group at C3 position, thus filling the gaps between phospholipid molecules and regulating the properties of liposomal membranes. To verify the calculation, membrane fluidity and micro-polarity of Lp, C-Lp and Rg3-Lp were measured, respectively. Membrane fluidity can reflect the regulatory role of the C17 side chain and planar ring structure of membrane regulator on the lipid bilayer, whereas membrane micro-polarity can reflect the hydrogen bond interaction between the C3 hydroxyl group of the regulator and phospholipid molecules. Consistent with MD calculation, Rg3-Lp showed a comparable effect to C-Lp in membrane fluidity enhancement since Rg3 and cholesterol are similar in their C17 side chain and planar ring structure. Meanwhile, Rg3-Lp exhibited stronger micro-polarity than Lp and C-Lp because of the intensive hydrogen bonds formed between its glycosyl units and the polar head of phospholipids, which further constituted hydrogen bond networks to stabilize the entire system. Therefore, Rg3-Lp/DTX showed improved leakage stability compared to that of C-Lp/DTX. In addition, MD simulation results also confirmed that the glycosyl moieties of Rg3 could stick out of the liposome surface, endowing Rg3-Lp with the potential to recognize and interact with Glut1. Molecular docking results showed that Rg3 can intensively hydrogen-bonded to Glut1 via its glycosyl moieties, while cholesterol failed due to the lack of glycosyl units, which implies that Rg3 has great potential as a targeting membrane material for tumor-specific delivery through its surface glycosyl moieties exposed on liposome surface. Cellular uptake of Rg3-Lp on 4T1 cells and the in vivo imaging of Rg3-Lp distribution proved that much more Rg3-Lp was uptake by 4T1 cells than C-Lp, and Rg3-Lp can accumulate more at tumor site than C-Lp. After knocking down of Glut1 gene in 4T1 cells, cellular uptake of Rg3-Lp was reduced to a level comparable to that of C-Lp, suggesting that Rg3-Lp can be preferentially uptake by tumor cells via the interaction between Rg3’s glycosyl units extending outside and Glut1 overexpressed on tumor cells, thereby realizing more tumor site accumulation than C-Lp.

However, the clinical therapeutic efficacy of active targeting liposomes is still compromised by the mechanical desmoplastic barrier and cold tumor immunity of TNBC [53, 54]. The dense stroma cells act as a physical barrier against infiltration of immune cells and the liposomes. There is a positive link between desmoplasia with impaired tumor immunity and limited efficacy of liposomal delivery [55]. Therefore, researchers have combined TEM remodeling strategies with liposomal chemotherapy. CAFs have been widely concerned as a TEM remodeling target due to the fact that desmoplasia is derived mainly from CAFs, the largest component of the stroma cells [56]. Consequently, some researchers applied two-stage therapy for better cancer treatment (first stage for anti-CAFs drug-loaded nanodrugs administration, second stage for antitumor drug-loaded nanodrugs administration) [24, 25]. However, such sequential targeting treatment of CAFs and tumor cells inevitably involve the decoration with targeting ligands for CAFs and tumor cells, respectively. Such overcomplicated formulation processes would pose difficulties for scale-up production and clinical translation.

To solve the problem, the interaction between tumor and CAFs was investigated in our study. Cytokines are critical mediators of the crosstalk between tumor cells and their surrounding TMEs [57]. We found that the expression of a-SMA, a hallmark of CAFs, was highly enhanced in 3T3 cells when cultured with 4T1 conditioned medium (4T1-CM), and was decreased distinctly when SB-431542, a TGF-*β* receptor inhibitor, was added in 4T1-CM. It demonstrated that TGF-*β* secreted by 4T1 cells was essential for facilitating the conversion of normal fibroblasts into CAFs. The finding is consistent with the previous studies showing that TGF-*β* secreted from tumor cells can activate CAFs through irritating TGF-*β* receptor 1 [58]. Moreover, in our study, Rg3 was proved to inhibit tumor cells’ ability to activate CAFs through regulating the TGF-*β* secretion by tumor cells. In ELISA assays, the level of TGF-*β* secreted form 4T1 cells in 4T1-CM was significantly decreased when pretreated with Rg3 (4T1-CM@Rg3). In addition, p-Smad2/3 and a-SMA expression in 3T3 cells was obviously lower in 4T1-CM@Rg3 group than those in 4T1-CM group. It means that Rg3 could effectively inhibit the activation of CAFs by suppressing the interaction between tumor and CAFs. Briefly, Rg3 is potential as an adjuvant agent combined with chemotherapy for TNBC that can prevent tumor cells from educating CAFs activation as well as promote cytotoxic efficacy of DTX. Therefore, unlike previous strategies, we only need to integrate Rg3 and DTX into a single tumor cell targeting liposome instead of the sequential targeting therapy for CAFs and tumor cells since Rg3 itself could reverse CAFs to resident stage by modulating tumor TGF-*β* secretion.

Owing to the excellent delivery efficiency of Rg3-Lp proved above, lower levels of TGF-*β* and α-SMA expression were detected in tumor tissue in vivo in Rg3-Lp group than those in Rg3 group in ELISA, WB and flow cytometry assays. Combined with these results, our study demonstrated that Rg3 can inhibit the tumor cells-mediated CAFs activation as a TME remodeling drug and realize tumor targeting delivery as a targeting liposome membrane material simultaneously. With the decrease of activated CAFs in tumor tissues, Rg3-Lp/DTX was proved to penetrate deeper into tumor tissues by 3D stroma-rich tumor spheroid penetration assay and fully observation of the α-SMA and TUNNEL signals in tumor slices. Subsequently, immunosuppressive TME was reversed by Rg3-Lp/DTX. More CD8^+^ T cells and antitumor M1 phenotype infiltrated in tumors in Rg3-Lp/DTX group, turning the TME from “cold” to “hot”. Collectively, Rg3-Lp/DTX showed excellent anti-TNBC efficacy, even better than the marketed DTX nano-formulation—Nanoxel-PM.

Herein, we designed one smart and versatile Rg3 liposome loading with DTX to achieve active tumor targeting and TME remodeling without any synthesis processes. The liposome was just composed of Rg3, phospholipids and DTX, making it very easy to prepare. Most importantly, Rg3-Lp/DTX achieved excellent tumor inhibition effect compared with Nanoxel-PM, the marketed micelles of DTX, with Rg3 as a targeting liposome material, an adjuvant with DTX and a TEM remodeling drug. The formulation is under pre-clinical studies now and of great potential to provide an effective drug for clinical treatment of TNBC.

## Conclusion

In summary, we successfully developed a multifunctional Rg3 liposome loading with DTX. The substitution of cholesterol with Rg3 endowed the liposome with the active targeting capacity for Glut1 overexpressed in TNBC tumor cells. Therefore, Rg3-Lp/DTX accumulated more at tumor site compared with C-Lp/DTX. After delivered to tumor cells, TGF-*β* secretion was inhibited by Rg3, which hindered tumor cells from educating resident cells into CAFs via suppressing TGF-*β*/Smad signaling. Decreased CAFs levels in tumor led to deeper liposome penetration and activated tumor immune microenvironment. Therefore, Rg3-Lp/DTX significantly retarded the TNBC growth. It can be concluded that Rg3-Lp/DTX can achieve tumor cell targeting and cold–hot tumors transformation via Rg3, thereby improving the therapeutic effect of loaded DTX. The application of the versatile liposomal system can be readily extended to other stroma-rich cancers, such as pancreatic, prostate, ovarian, and colon cancers. Thus, this study provides a smart and simple strategy with great clinical prospects for effective cancer treatment.

## Methods

### Materials

Cholesterol was obtained from Sinopharm Chemical Reagent Co., Ltd. (China); Lecithin PL-100 M was obtained from AVT Pharmaceutical Co., Ltd. (China); Ginsenoside 20(S)-Rg3 and Nanoxel-PM (Samyang Biopharm) were provided by Xiamen Ginposome Pharmatech Co., Ltd. (China); WZB117 was obtained from Medchem Express (USA); Coumarin 6 (C6) was purchased from Aladdin reagent Co., Ltd.. (China); Docetaxel and propidium iodide (PI) were obtained from Meilunbio Co., Ltd. (China).

### Cell culture

4T1 cells were acquired from Cell Bank of Shanghai, Chinese Academy of Sciences (China) and cultured in RPMI 1640 medium supplemented with 10% fetal bovine serum, 100 U/mL penicillin and 100 mg/mL streptomycin.

### Animals

Female BALB/c mice (6–8 weeks) were purchased from Shanghai SLAC Laboratory Animal Co., Ltd. All animals were treated according to the Guide for the Care and Laboratory Animals and all experiments were approved and performed according to the guidelines of the Ethics Committee of Fudan University (certificate number: 2020-04-YJ-WJX-01).

### Preparation and characterization of liposomes

The formulation in which PL-100 M/ Rg3 (or cholesterol)/docetaxel dissolved at the weight ratio of 10:3:1 in the organic solution (chloroform: ethanol 1:1) was prepared. The obtained solution was subjected to rotary evaporation to form thin films at 48 ℃ and then the films were hydrated with PBS (pH 7.4) at 48 ℃ for 30 min. Rg3-Lp/DTX was then obtained by ultrasonicating the suspension with a ultrasonicator (JYD-650, Zhixin Instrument Co., Ltd. Shanghai, China). Except for the initial addition of the dye solution (coumarin 6 (C6) or DiD, 5 mg/ml, 10 μl) to the organic solvent, the preparation of fluorescent labeled liposomes were prepared with the same process as described above. The particle size and zeta potential of C-Lp/DTX and Rg3-Lp/DTX were determined on a Zetasizer (Malvern, UK). Meanwhile, the morphology of the DTX-loaded liposomes was observed with a transmission electron microscopy (TEM, Tecnai G2 F20 S-Twin, FEI, USA).

To examine storage stability of liposomes, the prepared liposomes were stored at 4 ℃ for 7 days. The size and leakage of C-Lp/DTX and Rg3-Lp/DTX were measured every day for seven days. The mean size of the liposomes stored at 4 ℃ were measured by DLS every day. The DTX content remaining as loaded in liposomes was tested every day to determine the change of encapsulation efficacy of DTX-loaded liposomes during the storage period using high performance liquid chromatography (HPLC) and the leakage percentage of DTX was obtained according to the following equation.

$$\mathrm{DTX \, leakage \, percentage }\left(\mathrm{\%}\right)=\left(1-\frac{{W}_{\mathrm{DTX \, encapsulated  \, in \, liposome}}}{{W}_{Total \, DTX}}\right)\times 100\%$$

### Molecular dynamic simulation of Rg3-Lp

A 700-ns dynamic simulations at a time step of 2 fs were applied to establish the initial system (DSPC:Rg3:water molecules = 128:6:10,484). Then, a larger mixed bilayer system consisting of 300 DSPC and 90 Rg3 molecules was generated with memgen tool. a total of 300 ns simulation was conducted for the system. The CHARMM36 force field was applied through MD simulations,. NPT ensemble was applied for the simulation with the Nose–Hoover thermostat to keep the temperature at 300 K and the Parrinello–Rhaman method to maintain a constant pressure of 1 atm. LINCS algorithm was applied for the constraint of H-bonds lengths. Partical-Mesh Ewald (PME) method was utilized to calculate the long-rang electrostatic interactions with PME grid of 0.12 nm in the reciprocal-apace interactions and cubic interpolation. The cut-off distance for the long-range neighbor list of electrostatic and van der Waals interactions was 12 Å. Then, the obtained simulation system was visualized with the visual molecular dynamics (VMD) software.

### Liposome membrane micro-polarity measurement

0.1 ml of the 4 × 10^−7^ M tritium solution was added in 10 ml tubes and its organic solvent was evaporated overnight. Then we added 5 ml different liposome solution respectively and sonicated them for 10 min. The mixture was left for 12 h at room temperature. The fluorescence intensities at 373 nm (*I*_1_) and 384 nm (*I*_3_) which were excited at 338 nm was recorded, respectively. The value of *I*_1_/*I*_3_ could reflect the microenvironmental polarity of the liposomal membranes.

### Liposome membrane fluidity measurement

One milliliter of the 2 × 10^−6^ M DPH solution was mixed with 5 ml different liposomes, respectively. The fluorescence intensity of the mixture was recorded (Ex/Em = 360 nm/430 nm), respectively after leaving the mixture at room temperature for 12 h. The degree of polarization was obtained from the following formula:

$$P=\frac{{F}_{\| }-\mathrm{G}{F}_{\perp }}{{F}_{\| }+\mathrm{G}{F}_{\perp }}$$

F_‖_ and F_⊥_: the fluorescence intensities of the emitted light polarized parallel and perpendicular to the polarized light of excitation; G: the grating correction factor. The value of the polarization of DPH represented the membrane fluidity. The higher the P value, the lower the membrane fluidity.

### Molecular docking of Glut1-Chol and Glut1-Rg3

The structure of ginsenoside Rg3 and cholesterol (Chol) were constructed with Chemdraw and Chem3D was applied to converted them into three-dimensional structures. The 3D structure of Glut1 was obtained from a protein data bank with a PDB number 4PYP. Then, we imported the structure of Glut1, Chol and Rg3 in Schrödinger maestro version 11.8. After ligand preparation, all possible conformations of Rg3 or Chol were developed. Then, each conformation of the ligands was docked to Glut1 and results were ranked with a docking score. The optimal docking conformation was determined based on the docking score and hydrogen bonding interactions. PyMol was utilized to generate the ribbon/surface view of docked complexes.

### Intracellular uptake assay of the liposomes by 4T1 cells

2 × 10^5^ 4T1 cells were seeded in 12-well plates per well. In Rg3-Lp/C6 + glucose, Rg3-Lp/C6 + WZB117 groups, the medium was aspired after 12 h. 20 mM glucose solution and 10 μM WZB117 was added and incubated with cells for 1 h, respectively. The cells were then treated with C-Lp loaded with C6 (C-Lp/C6) and Rg3-Lp loaded with C6 (Rg3-Lp/C6) respectively (C6 100 ng/mL) for 4 h. The cells were then collected, washed three times with pre-chilled PBS and analyzed by flow cytometry (CytoFlex S, Beckman Coulter, Inc., USA).

### Intracellular uptake of liposomes by Glut1-knockdown 4T1 cells

Glut 1 siRNA (5’- CCAACUGGACCUCAAACUUTT -3’) and siRNA mate were mixed in opti-MEM medium to form siRNA complexes. Then siRNA complex was added in cell culture medium and incubated with 4T1 cells for 72 h. Then, C-Lp/C6 and Rg3-Lp/C6 was added in the medium respectively for 4 h (C6 100 ng/mL). The cells were prepared and analyzed by flow cytometry (CytoFlex S, Beckman Coulter, Inc., USA). For confocal laser scanning microscope (CLSM) observation, the cells were immunofluorescence (IF) stained and imaged as described previously [31].

### In vitro cytotoxicity studies

The cytotoxicity of free DTX and different DTX formulations against 4T1 cells was examined with MTT cytotoxicity assay. 5 × 10^3^ 4T1 cells were seeded per well in 96-well plates. After 12 h, DTX, C-Lp/DTX, Nanoxel-PM, Rg3, Rg3-Lp, mixture of Rg3 and DTX (Rg3/DTX) and Rg3-Lp/DTX within a certain DTX concentration range was added in the medium and incubated with 4T1 cells for 48 h, respectively. 50 μL of MTT (2 mg/mL) was then added and incubated for another 4 h. Then, the medium was aspirated and 200 μL DMSO was added. After shaking for 30 min, the optical density (OD) value of each well was measured at 490 nm using a microplate reader (Tecan Trading Co., Ltd., Switzerland). The cell viability rate was calculated according to the following formula:

$$\mathrm{Cell \, viability \, rate} (\%)= \frac{({\mathrm{OD}}_{\mathrm{sample}}-{\mathrm{OD}}_{\mathrm{blank}} )}{({\mathrm{OD}}_{\mathrm{Control}}-{\mathrm{OD}}_{\mathrm{blank}} )} \times 100\mathrm{\%}$$

Control: Untreated cells (viability rate 100%); Blank: (the wells with no cells).

### Cell apoptosis assay

1 × 10^5^ 4T1 cells were seeded in 12-well plates per well. DTX, C-Lp/DTX, Nanoxel-PM, Rg3, Rg3-Lp, Rg3/DTX and Rg3-Lp/DTX was added in the medium and incubated with 4T1 cells, respectively (DTX 0.5 μg/mL). Then the cells were collected and washed with PBS. Next, the cells were further stained with propidium iodide (PI) and Annexin V-FITC in binding buffer for 15 min at room temperature. The apoptosis rate of the cells was then analyzed using a flow cytometer (BD Biosciences, USA).

At the same time, the 4T1 cells suspension (2 × 10^5^/ mL) was inoculated into 12-well plate with prepared cell sheets (0.1 ml cell suspension every cell sheet). The drug solution was changed according to the group described before after 24 h. The nuclei were stained with Hoechst 33,342 and PI after 48 h of induction, then mounted with glycerol jelly mounting medium and exposed to inverted fluorescence microscope (Leica, DMI4000D, Germany) for qualitative observation and photo taking.

### Culture and polarization induction of mouse bone marrow-derived macrophage (BMDM)

Bone marrow cells were collected from 6–8-week old specific pathogen-free male Balb/c mice as described above [29]. Bone marrow cells were rinsed with serum-free DMEM and cultured in fresh DMEM containing 20 ng/mL macrophage colony-stimulating factor for 96 h to induce bone marrow derived macrophages (BMDM) differentiation. Interferon-γ (20 ng/mL) and lipopolysaccharide (LPS, 500 ng/mL) were added to the medium for 24 h to induce the polarization toward M1 phenotype.

1 × 10^5^ 4T1 cells were incubated in a 6-well plate per well and treated overnight with PBS, DTX, C-Lp/DTX, Nanoxel-PM, Rg3, Rg3-Lp, Rg3/DTX and Rg3-Lp/DTX respectively (DTX 5 μg/mL). 4T1-cultured medium after different treatment was then collected. Then, 1 × 10^5^ 3T3 cells were incubated in a 6-well plate per well and treated for 24 h with 4T1-CM plus SB-431542 (MCE, USA), a kind of TGF-*β* inhibitor or with different 4T1 cultured medium collected from 4T1 cells after treatment with PBS (4T1-CM), DTX (4T1-CM@DTX), C-Lp/DTX (4T1-CM@C-Lp/DTX), Nanoxel-PM (4T1-CM@Nanoxel-PM), Rg3 (4T1-CM@Rg3), Rg3-Lp (4T1-CM@Rg3-Lp), Rg3/DTX (4T1-CM@Rg3/DTX) or Rg3-Lp/DTX (4T1-CM@Rg3-Lp/DTX). 3T3 cultured medium collected from 3T3 cells pretreated with PBS (3T3-CM), 4T1-CM (3T3-CM @ 4T1-CM), 4T1-CM/SB-431542 (3T3-CM@(4T1-CM/SB-431542)), 4T1-CM@DTX (3T3-CM@ (4T1-CM@DTX)), 4T1-CM@C-Lp/DTX (3T3-CM@ (4T1-CM@C-Lp/DTX)), 4T1-CM@Nanoxel-PM (3T3-CM@(4T1-CM@Nanoxel-PM)), 4T1-CM@Rg3 (3T3-CM@(4T1-CM@Rg3)), 4T1-CM@Rg3-Lp (3T3-CM@(4T1-CM@Rg3-Lp)), 4T1-CM@Rg3/DTX (3T3-CM@(4T1-CM@Rg3/DTX)) or 4T1-CM@Rg3-Lp/DTX (3T3-CM@(4T1-CM@Rg3-Lp/DTX)) were collected. The M1-type macrophages were incubated with different conditioned 3T3 cultured medium. After 48 h, the cells were collected and incubated with APC-F4/80 (BioLegend, UK), FITC-CD206 (BioLegend, UK) and PE-CD86 (BioLegend, UK) antibodies to label M2 and M1 cells, respectively. Then, the cells were analyzed by flow cytometry (CytoFlex S, Beckman Coulter, Inc., USA).

### Penetration assay of the liposomes in 3D stroma-rich tumor spheroids

Unlike the spontaneous process of enriching fibroblasts by tumor cells during tumor progression in vivo, tumor cells were mixed with fibroblasts NIH-3T3 cells in vitro to manually mimic the stroma-rich TME [46]. 3D stroma-rich tumor spheroids containing 4T1 and NIH-3T3 were generated. 0.6 g of agarose was weighed and added to 30 mL of serum-free DMEM medium containing 1% cyan-chain double antibody, and kept in a constant temperature water bath at 80 °C for 30 min, then transferred to the autoclave at 121 °C. Sterilize under high pressure for 30 min. After sterilization, the agarose solution was added to a 96-well plate with 50 μL per well. The 4T1 cells and 3T3 fibroblasts were then digested, mixed and added to a 96-well cell culture plate containing agarose gel at 3 × 10^3^ cells/100 μL and 1 × 10^3^ cells/100 μL per well respectively. It was placed in a 37° C incubator for cultivation. The fluid was changed every other day, and the tumor sphere grew to about 500 μm after 10 days. C-Lp/C6 and Rg3-Lp/C6 were then administered, respectively. After incubating for 12 h, the tumor spheres were washed three times with PBS buffered saline solution, then transferred to a small dish, fluorescence of C6 was observed with CLSM (Leica, DMI4000D, Germany) from the top layer of the tumor sphere to the bottom layer, a tomographic scan is performed every 10 μm. After the scanning, the penetration depth of the nanoparticles is analyzed and counted by the ZEN software provided by the instrument.

### Immunofluorescence staining of α-SMA

1 × 10^5^ 3T3 cells were incubated in a 6-well plate per well and treated overnight with TGF-*β* (20 ng/ml), TGF-*β* (20 ng/ml) plus SB-431542 (MCE, USA), a kind of TGF-*β* inhibitor, different 4T1 cultured medium after treatment with PBS (4T1-CM), DTX (4T1-CM@DTX), C-Lp/DTX (4T1-CM@C-Lp/DTX), Nanoxel-PM (4T1-CM@Nanoxel-PM), Rg3 (4T1-CM@Rg3), Rg3-Lp (4T1-CM@Rg3-Lp), Rg3/DTX (4T1-CM@Rg3/DTX) or Rg3-Lp/DTX (4T1-CM@Rg3-Lp/DTX) (DTX concentration, 0.5 μg/mL), respectively. 4T1-conditioned medium after different treatment was obtained for Enzyme-linked immunosorbent assay (ELISA assay) after 24 h. The 3T3 cells after different treatment were then IF stained and imaged with primary anti-α-SMA (ab124964, Abcam) and Cy3-labeled fluorescent secondary antibody (33108ES60, Yeasen) according to the procedure as described before [32].

### In vivo imaging of tumor bearing mice

5 × 10^5^ 4T1 cells were orthotopically injected into a mammary fat pad in the lower right quadrant of the abdomen of Balb/c female mice to develop the orthotopic TNBC model. Treatment was initiated at about 7 days after inoculation. The tumor bearing mice were divided randomly into two groups and were injected intravenously with C-Lp/DiD and Rg3-Lp/DiD respectively. In vivo fluorescent images were taken under in vivo imaging system (IVIS) at 1, 2, 4, 8, 12 and 24 h after injection. After 24 h, the mice were killed. Tumors, hearts, livers, spleens, lungs and kidneys were then collected and imaged under IVIS system.

### In vivo antitumor efficacy

Orthotopic TNBC model was developed by injecting 4T1 cells into a mammary fat pad in the lower right quadrant of the abdomen of BALB/c female mice. After 7 days, the mice were divided randomly into 8 groups (n = 6 per group) and each treated group was injected intravenously with PBS, DTX, C-Lp/DTX, Nanoxel-PM, Rg3, Rg3-Lp, Rg3/DTX and Rg3-Lp/DTX (10 mg/kg of DTX) every 4 days for 20 days respectively. The length and width of tumors and the body weight were measured simultaneously. The tumor volume (V) was calculated using the following formula:

$$\mathrm{V}=\frac{\left({W}^{2}\times \mathrm{L}\right)}{2}$$

Length (L) is the longest diameter and width (W) is the shortest diameter perpendicular to length. At Day 20, all mice were sacrificed, and their tumors were harvested for photo imaging and histological examination. For the histological analysis of apoptosis cells and CAFs in tumor tissue, the TdT-mediated dUTP Nick-End Labeling (TUNEL) assay and α-SMA staining of tumor slices was performed and full-scanned, In addition, for the histological analysis of the collagen in tumor tissue, MASSON staining was conducted and five randomly chosen microscopic fields were selected and semi-quantified by ImageJ software.

### Enzyme-linked immunosorbent (ELISA) assay

1 × 10^5^ 4T1 cells were incubated in a 6-well plate per well and treated overnight with PBS, DTX, C-Lp/DTX, Nanoxel-PM, Rg3, Rg3-Lp, Rg3/DTX and Rg3-Lp/DTX respectively (DTX 5 μg/mL). 4T1-CM after different treatment was then collected. The concentration of TGF-*β* in the different medium was detected with ELISA kit (Neobioscience Co., Ltd., China) according to manufacturer's instruction. The results were read using a microplate spectrophotometer at 450 nm (Thermo Multiskan MK3, USA). Tumor tissues were obtained according to the protocol in In vivo antitumor efficacy and homogenized with pre-cooled PBS (5 ml PBS/1 g tumor). The prepared homogenate was centrifuged at 5000 g for 5 min and the TGF-*β* concentration in the supernatant was detected with the ELISA kit (Neobioscience Co., Ltd., China) according to manufacturer's instruction.

### Western Blot (WB) assay

3T3 cells were incubated in a 6-well plate at a density of 1 × 10^5^ cells per well and treated overnight with TGF-*β* (20 ng/ml), TGF-*β* (20 ng/ml) plus SB-431542 (MCE, USA), a kind of TGF-*β* inhibitor, different 4T1 cultured medium after treatment with PBS (4T1-CM), DTX (4T1@DTX), C-Lp/DTX (4T1@C-Lp/DTX), Nanoxel-PM (4T1@Nanoxel-PM), Rg3 (4T1@Rg3), Rg3-Lp (4T1@Rg3-Lp), Rg3/DTX (4T1@Rg3/DTX) or Rg3-Lp/DTX (4T1@Rg3-Lp/DTX) (DTX 0.5 μg/mL). The protein of the cells was then harvested according to the procedures described previously [32]. 50 mg of protein per lane were loaded on the polyacrylamide gel and then transferred onto a PVDF membrane. The PVDF membrane was incubated with anti-α-SMA (ab124964, Abcam), anti-phospho-Smad2/3 (ab272332, Abcam) and anti-GAPDH (30202ES40, Yeasen) overnight at 4℃ respectively. The following procedure was performed as previously described [32].

### Quantification of tumor-infiltrating lymphocytes

Tumor tissues were obtained according to the protocol in In vivo antitumor efficacy. Cell suspensions derived from the obtained tumor tissues were prepared by grinding tumor tissues and passing the homogenate through 200-mesh sieve. The cell suspensions were then co-incubated with antibodies for T cells staining (CD45, CD4 and CD8), myeloid-derived suppressor cells staining (Gr1, CD11b and CD45), Tregs staining (CD45, CD4 and Foxp3), macrophages staining(CD45, F4/80, CD86 or CD206) and CAFs staining (α-SMA) for FACS analysis (BD Biosciences, USA).

### Quantitative PCR (q-PCR) analysis

Total RNAs were extracted with Trizol from tumor tissues obtained following the protocol of Quantification of tumor-infiltrating lymphocyte. Quantitative real-time PCR analysis were performed according to the procedures described before [32]. The mouse α-SMA primer pairs were 5′- ACACGGCATCATCACCAACTG -3′ and 5′- TTGGCCTTAGGGTTCAGTGGTGTC-3′, The mouse GAPDH primer pairs were 5’- CCTCGTCCCGTAGACAAAATG-3′ and 5′-TGAGGTCAATGAAGGGGTCGT-3′.

### Safety evaluation

Tumor bearing mice were randomly divided into 8 groups. The administration protocol was the same as described above. At the end point, after the mice were sacrificed, their main organs (heart, liver, spleen, lung, kidney) were excised and weighted for the calculation of organ weight index, then treated for histological examination. Blood samples were also collected for routine blood analyses.

$$\mathrm{Organ \, weight \, index}=\frac{{W}_{organ}}{{W}_{whole \, body}}\times 100\%$$

### Statistical analysis

Results are expressed as mean ± S.D. Statistical analysis was conducted with GraphPad Prism version 9.3.1. Two-tailed Student’s t test was applied for differences between two experimental groups; one-way analysis of variance (ANOVA) with Tukey’s post hoc test was carried out for differences among multiple groups. Statistically significance was considered at *p* < 0.05 (*p < 0.05, **p < 0.01, and ***p < 0.001, ns: no significant difference).

## Supplementary Information

**Additional file 1: Fig. S1.** The chemical structure of (A) cholesterol and (B) Rg3. **Fig. S2.** The size distribution of the Nanoxel-PM. **Fig. S3.** The size stability of the C-Lp/DTX and Rg3-Lp/DTX. **Fig. S4.** The quantitative analysis of cellular uptake of C-Lp/C6, Rg3-Lp/C6 and Rg3-Lp/C6 with GLUTs inhibitors in 4T1 cells via flow cytometry. **Fig. S5.** Confocal laser scanning microscope (CLSM) images of the cellular uptake of C-Lp/C6, Rg3-Lp/C6 and Rg3-Lp/C6 with GLUT1 inhibitors in 4T1 cells. Scale bars: 20 μm. **Fig. S6.** Ex vivo imaging (A) and ROI values (B) of excised organs 24 h after injection. **Fig. S7.** Ratio of organ weight to body weight in 4T1-bearing mice at the end point of the treatment. n = 6 in each group. **Fig. S8.** Blood hematology tests were performed in tumor bearing mice with different treatment. **Fig. S9.** Quantitative western blot analysis of the level of p-Smad2/3 and α-SMA in 3T3 after different treatments. **Fig. S10.** Quantitative western blot analysis of the level of p-Smad2/3 and α-SMA in tumor tissues after different treatments. **Fig. S11.** Flow cytometry analysis of activated CAFs in tumor after treatment with PBS, DTX, C-Lp/DTX, Nanoxel-PM, Rg3, Rg3-Lp, Rg3/DTX and Rg3-Lp/DTX, respectively. **Fig. S12.** Representative gating strategy used for flow cytometry analysis of CD4^+^ T cells, CD8^+^ T cells, MDSC (CD11b^+^Gr1^+^), M1 macrophages (F4/80^+^CD86^+^), M2 macrophages (F4/80^+^CD206^+^), and Treg cells (CD4^+^Foxp3^+^) in the tumors (gated on CD45^+^ cells) after different treatments. **Fig. S13.** Flow cytometric and histogram analysis of M1-type and M2-type macrophages after different treatment. **Fig. S14.** Analysis of the level of collagens in tumors treated with PBS and different DTX formulations. **Fig. S15.** H&E staining of major organs.

## Data Availability

The authors declare that the main data supporting the findings of this study are available within the article and its Additional file Information. Extra data are available from the corresponding author upon request.

## Abbreviations

DTX: Docetaxel
TNBC: Triple negative breast cancer
TME: Tumor microenvironment
CAFs: Cancer-associated fibroblasts
Rg3/DTX: The mixture of Rg3 and DTX
Rg3-Lp/DTX: DTX-loaded Rg3 liposome
Glut1: Glucose transporter-1
ECM: Dense extracellular matrix
a-SMA: A-smooth muscle actin
MSCs: Mesenchymal stem cells
TGF-β: Transforming growth factor beta
IL-6: Interleukin-6
IL-10: Interleukin-10
CXCL12: C-X-C motif chemokine ligand 12
MTT: Thiazolyl Blue Tetrazolium Bromide
PI: Propidium iodide
TEM: Transmission electron microscope
HPLC: High performance liquid chromatography
C6: Coumarin 6
C-Lp/DTX: DTX loaded cholesterol liposomes
IVIS: In vivo imaging system
4T1-CM: 4T1-conditioned medium
ELISA: Enzyme-linked immunosorbent
PBS: Phosphate buffer saline
WB: Western Blot
q-PCR: Quantitative PCR
Rg3-Lp: Rg3 liposome
C-Lp: Cholesterol liposome
NFs: Normal fibroblasts
pSmad2/3: Phosphorylated Smad2/3
4T1-CM: 4T1 conditioned medium pretreated with PBS
4T1-CM@DTX: 4T1 conditioned medium pretreated with DTX
4T1-CM@C-Lp/DTX: 4T1 conditioned medium pretreated with C-Lp/DTX
4T1-CM@Nanoxel-PM: 4T1 conditioned medium pretreated with Nanoxel-PM
4T1-CM@Rg3: 4T1 conditioned medium pretreated with Rg3
4T1-CM@Rg3-Lp: 4T1 conditioned medium pretreated with Rg3-Lp
4T1-CM@Rg3/DTX: 4T1 conditioned medium pretreated with Rg3/DTX
4T1-CM@ Rg3-Lp/DTX: 4T1 conditioned medium pretreated with Rg3-Lp/DTX
G-MDSC: Granulocytic myeloid-derived suppressor cells
M-MDSC: Monocytic MDSC
