# Supplementary material for: Targeting therapy and tumor microenvironment remodeling of triple-negative breast cancer by ginsenoside Rg3 based liposomes
Source: J Nanobiotechnology. 2022 Sep 15;20:414. doi: 10.1186/s12951-022-01623-2 (PMC9479350; doi:10.1186/s12951-022-01623-2)
Supplement: Supplementary file 1 — Additional file 1: Fig. S1. The chemical structure of (A) cholesterol and (B) Rg3. Fig. S2. The size distribution of the Nanoxel-PM. Fig. S3. The size stability of the C-Lp/DTX and Rg3-Lp/DTX. Fig. S4. The quantitative analysis of cellular uptake of C-Lp/C6, Rg3-Lp/C6 and Rg3-Lp/C6 with GLUTs inhibitors in 4T1 cells via flow cytometry. Fig. S5. Confocal laser scanning microscope (CLSM) images of the cellular uptake of C-Lp/C6, Rg3-Lp/C6 and Rg3-Lp/C6 with GLUT1 inhibitors in 4T1 cells. Scale bars: 20 μm. Fig. S6. Ex vivo imaging (A) and ROI values (B) of excised organs 24 h after injection. Fig. S7. Ratio of organ weight to body weight in 4T1-bearing mice at the end point of the treatment. n = 6 in each group. Fig. S8. Blood hematology tests were performed in tumor bearing mice with different treatment. Fig. S9. Quantitative western blot analysis of the level of p-Smad2/3 and α-SMA in 3T3 after different treatments. Fig. S10. Quantitative western blot analysis of the level of p-Smad2/3 and α-SMA in tumor tissues after different treatments. Fig. S11. Flow cytometry analysis of activated CAFs in tumor after treatment with PBS, DTX, C-Lp/DTX, Nanoxel-PM, Rg3, Rg3-Lp, Rg3/DTX and Rg3-Lp/DTX, respectively. Fig. S12. Representative gating strategy used for flow cytometry analysis of CD4+ T cells, CD8+ T cells, MDSC (CD11b+Gr1+), M1 macrophages (F4/80+CD86+), M2 macrophages (F4/80+CD206+), and Treg cells (CD4+Foxp3+) in the tumors (gated on CD45+ cells) after different treatments. Fig. S13. Flow cytometric and histogram analysis of M1-type and M2-type macrophages after different treatment. Fig. S14. Analysis of the level of collagens in tumors treated with PBS and different DTX formulations. Fig. S15. H&E staining of major organs. [file 12951_2022_1623_MOESM1_ESM.docx]

Additional file information for

**Targeting Therapy and Tumor Microenvironment Remodeling of Triple-Negative Breast Cancer by Ginsenoside Rg3 Based Liposomes**

Jiaxuan Xia^1^, Shuya Zhang^1^, Ru Zhang^1^, Anni Wang^1^, Ying Zhu^2^, Meichen Dong^1^, Shaojie Ma^3^, Chao Hong^4^, Shengyao Liu^1^, Dan Wang^5^ and Jianxin Wang^1,6^*

^1^ Department of Pharmaceutics, School of Pharmacy, Fudan University & Key Laboratory of Smart Drug Delivery, Ministry of Education, Shanghai 201203, China

^2^ Department of Integrative Oncology, Fudan University Shanghai Cancer Center, Shanghai 200032, China

^3^ Key Laboratory of Molecular Biophysics of the Ministry of Education, College of Life Science and Technology, Huazhong University of Science and Technology, Wuhan 430071, China

^4^ Experiment Center of Teaching & Learning, Shanghai University of Traditional Chinese Medicine, Shanghai 201203, China

^5^ Xiamen Ginposome Pharmatech Co., Ltd, Xiamen 361026, People’s Republic of China

^6^ Institutes of Integrative Medicine, Fudan University, Shanghai 201203, People’s Republic of China

*Correspondence author: [jxwang@fudan.edu.cn](mailto:jxwang@fudan.edu.cn) (J. Wang).

**This PDF file includes:**

Figure S1. The chemical structure of (A) cholesterol and (B) Rg3.

Figure S2. The size distribution of the Nanoxel-PM.

Figure S3. The size stability of the C-Lp/DTX and Rg3-Lp/DTX.

Figure S4. The quantitative analysis of cellular uptake of C-Lp/C6, Rg3-Lp/C6 and Rg3-Lp/C6 with GLUTs inhibitors in 4T1 cells via flow cytometry.

Figure S5. Confocal laser scanning microscope (CLSM) images of the cellular uptake of C-Lp/C6, Rg3-Lp/C6 and Rg3-Lp/C6 with GLUT1 inhibitors in 4T1 cells. Scale bars: 20 μm.

Figure S6. *Ex vivo* imaging (A) and ROI values (B) of excised organs 24 h after injection.

Figure S7. Ratio of organ weight to body weight in 4T1-bearing mice at the end point of the treatment. n = 6 in each group.

Figure S8. Blood hematology tests were performed in tumor bearing mice with different treatment.

Figure S9. Quantitative western blot analysis of the level of p-Smad2/3 and α-SMA in 3T3 after different treatments (n=3 per group).

Figure S10. Quantitative western blot analysis of the level of p-Smad2/3 and α-SMA in tumor tissues after different treatments (n=3 per group).

Figure S11. Flow cytometry analysis of activated CAFs in tumor after treatment with PBS, DTX, C-Lp/DTX, Nanoxel-PM, Rg3, Rg3-Lp, Rg3/DTX and Rg3-Lp/DTX, respectively.

Figure S12. Representative gating strategy used for flow cytometry analysis of CD4^+^ T cells, CD8^+^ T cells, MDSC (CD11b^+^Gr1^+^), M1 macrophages (F4/80^+^CD86^+^), M2 macrophages (F4/80^+^CD206^+^), and Treg cells (CD4^+^Foxp3^+^) in the tumors (gated on CD45^+^ cells) after different treatments.

Figure S13. Flow cytometric and histogram analysis of M1-type and M2-type macrophages after different treatment (n=3 per group).

Figure S14. Analysis of the level of collagens in tumors treated with PBS and different DTX formulations.

Figure S15. H&E staining of major organs.

**
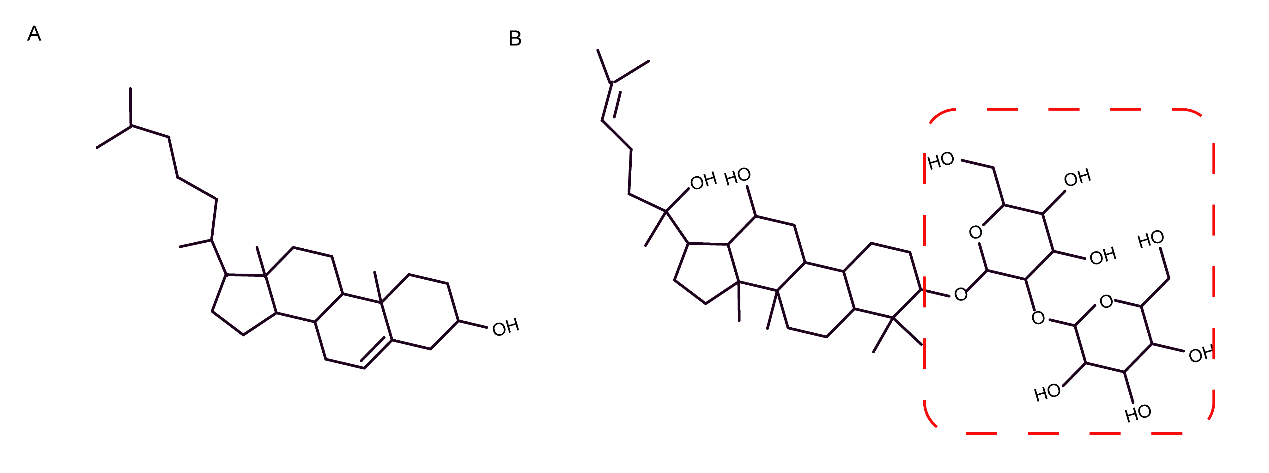
**

**Figure S1.** The chemical structure of (A) cholesterol and (B) Rg3


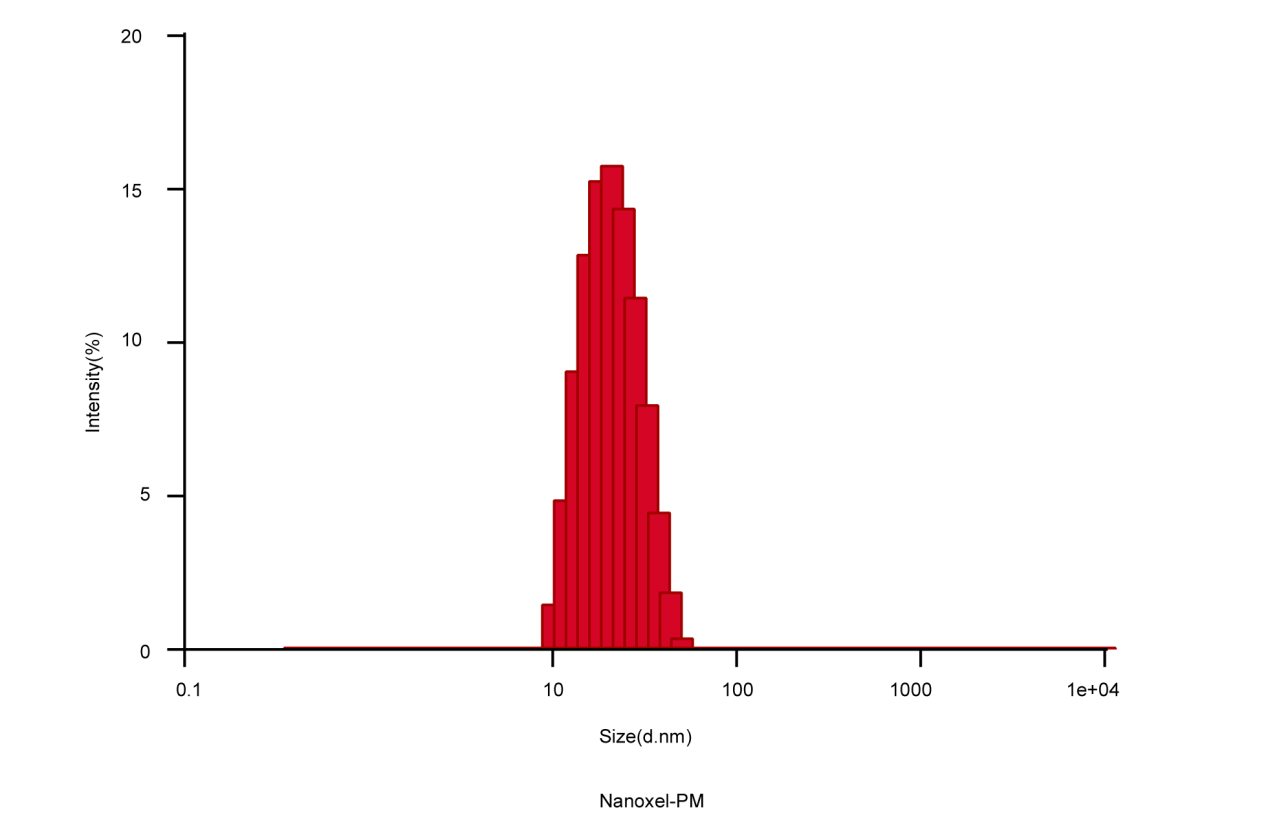


**Figure S2.** The size distribution of the Nanoxel-PM


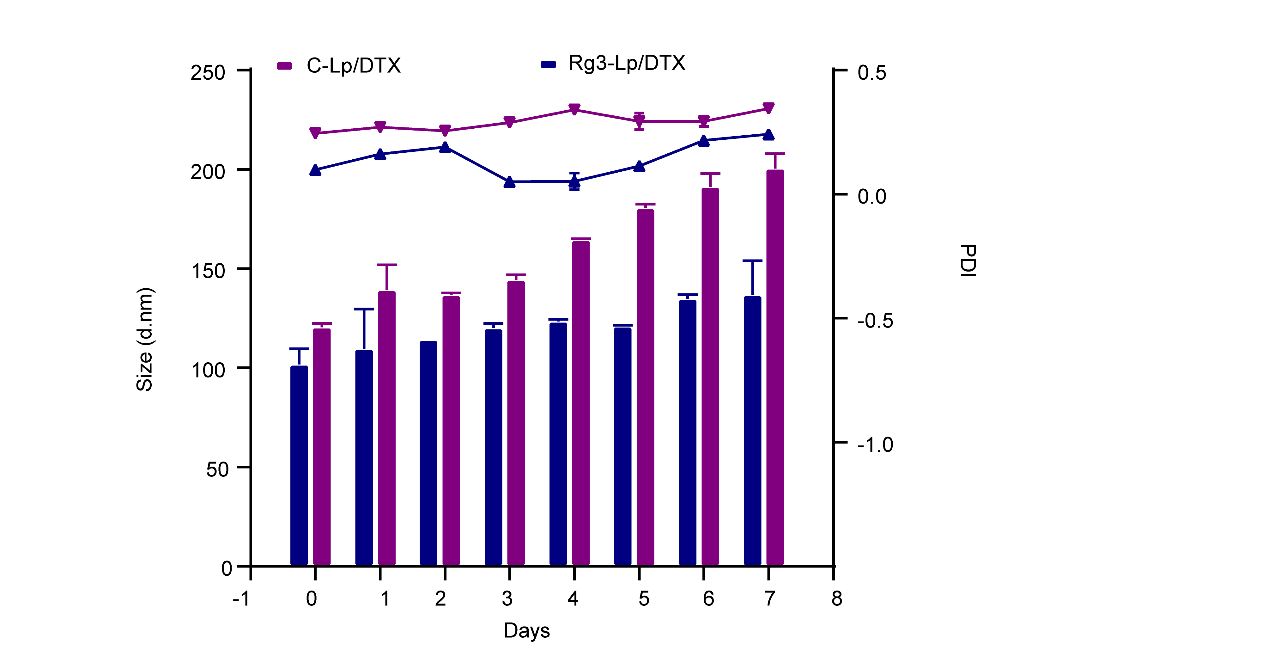


**Figure S3.** The size stability of the C-Lp/DTX and Rg3-Lp/DTX.

**
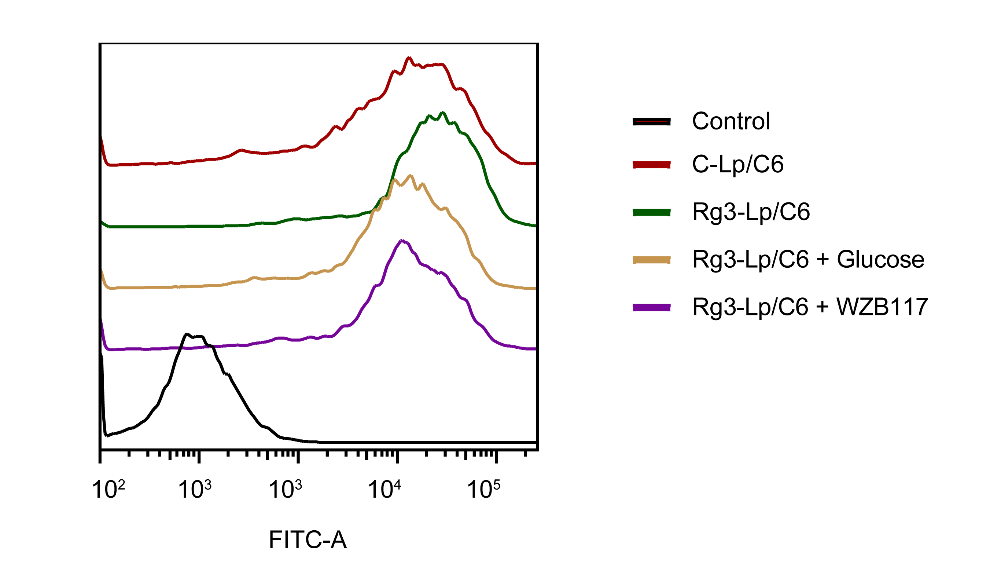
**

**Figure S4.** The quantitative analysis of cellular uptake of C-Lp/C6, Rg3-Lp/C6 and Rg3-Lp/C6 with GLUTs inhibitors in 4T1 cells via flow cytometry.


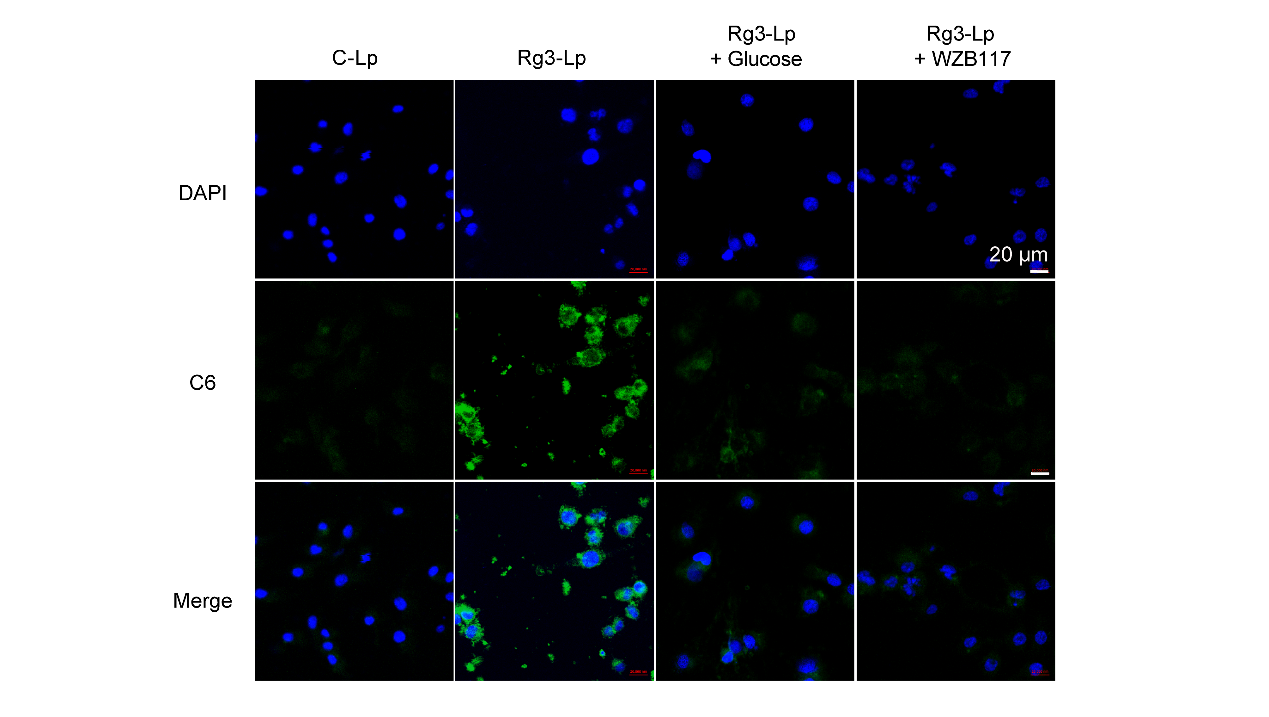


**Figure S5.** Confocal laser scanning microscope (CLSM) images of the cellular uptake of C-Lp/C6, Rg3-Lp/C6 and Rg3-Lp/C6 with Glut1 inhibitors in 4T1 cells. Scale bars: 20 μm.

**
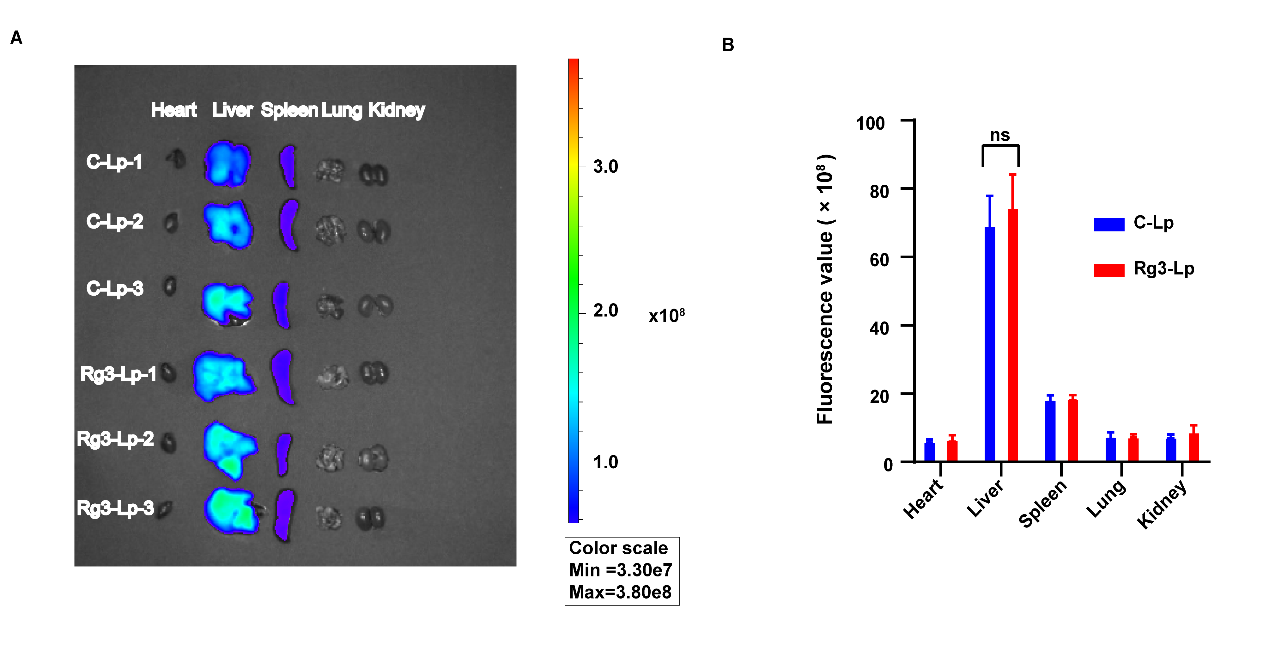
**

**Figure S6.** *Ex vivo* imaging (A) and ROI values (B) of excised organs 24 h after injection.


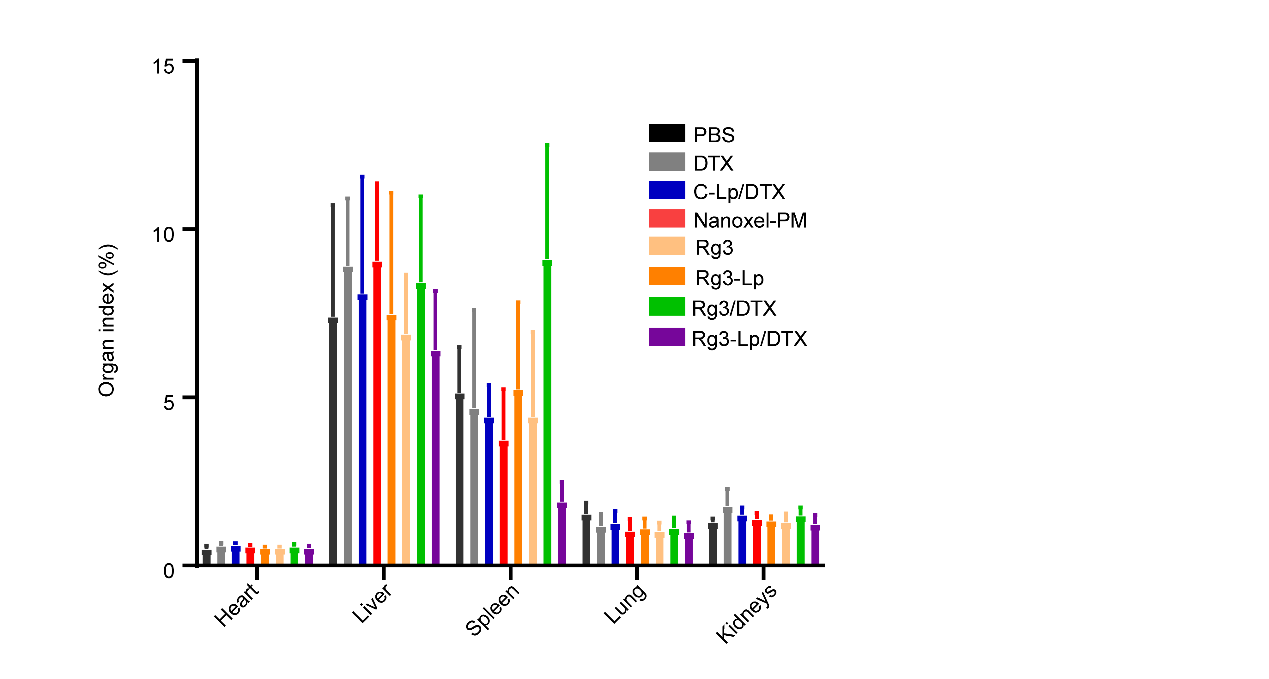


**Figure S7.** Ratio of organ weight to body weight in 4T1-bearing mice at the end point of the treatment. (n = 6)


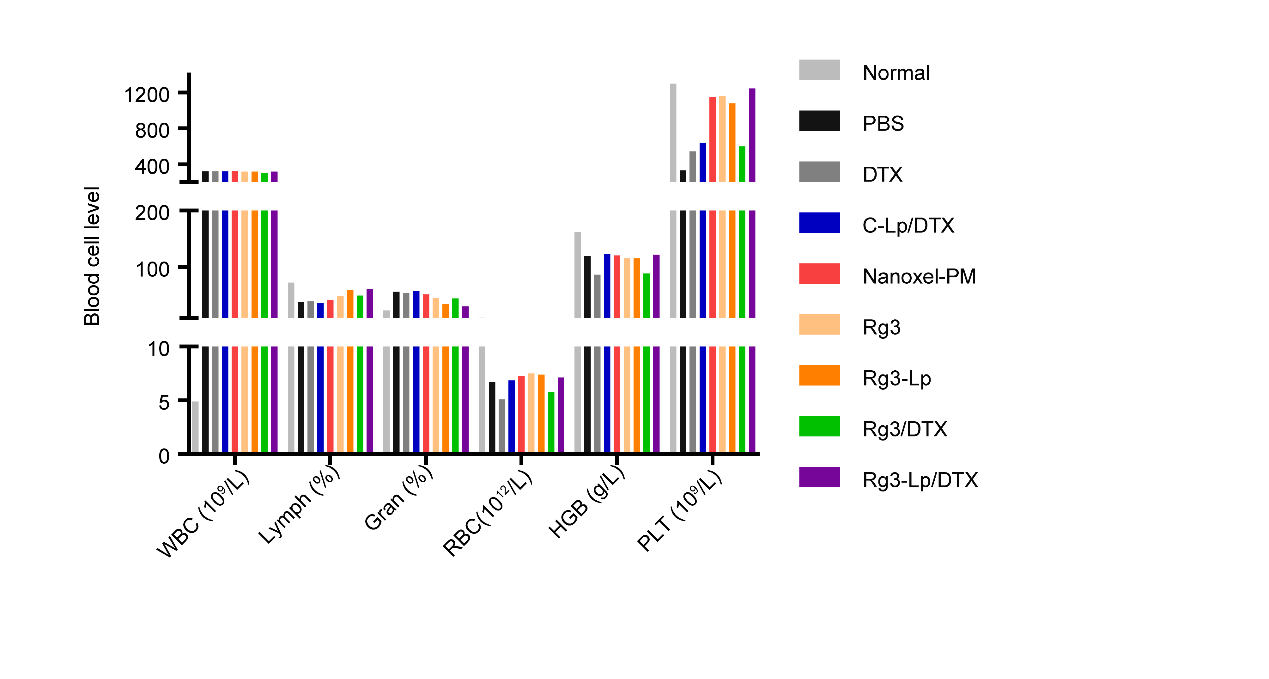


**Figure S8.** Blood hematology tests in tumor bearing mice with different treatment.


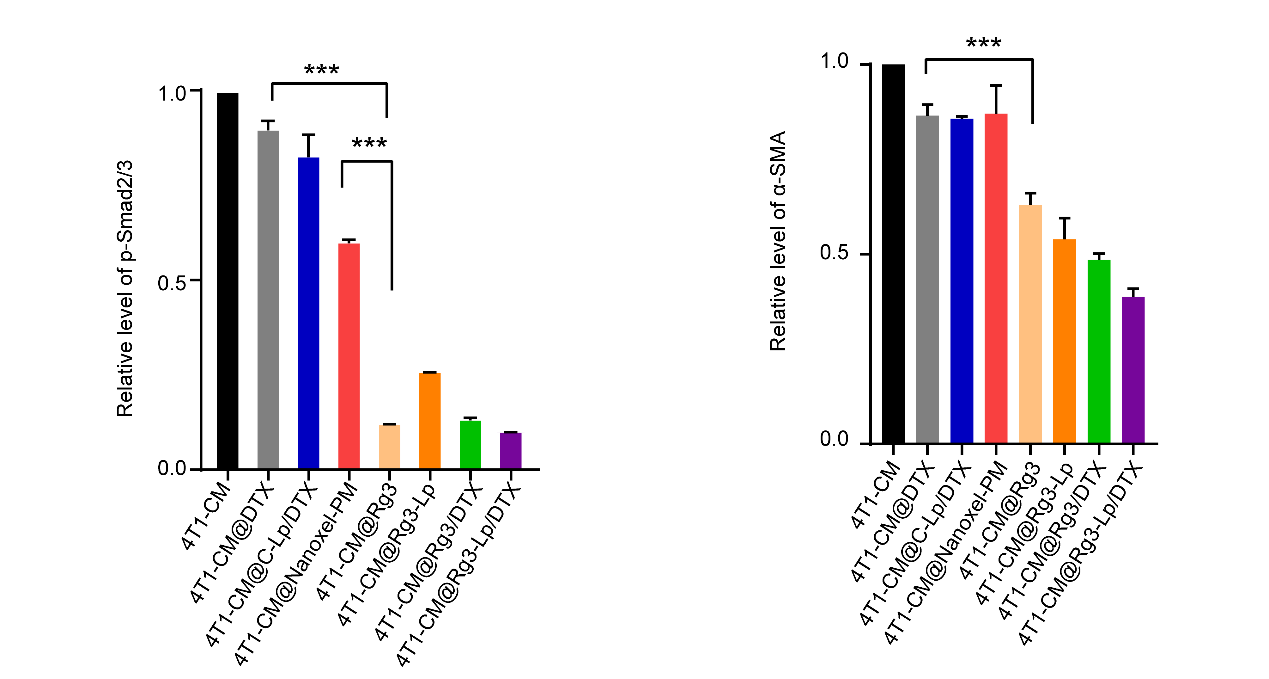


**Figure S9.** Quantitative western blot analysis of the level of p-Smad2/3 and α-SMA in 3T3 after different treatments (n=3).


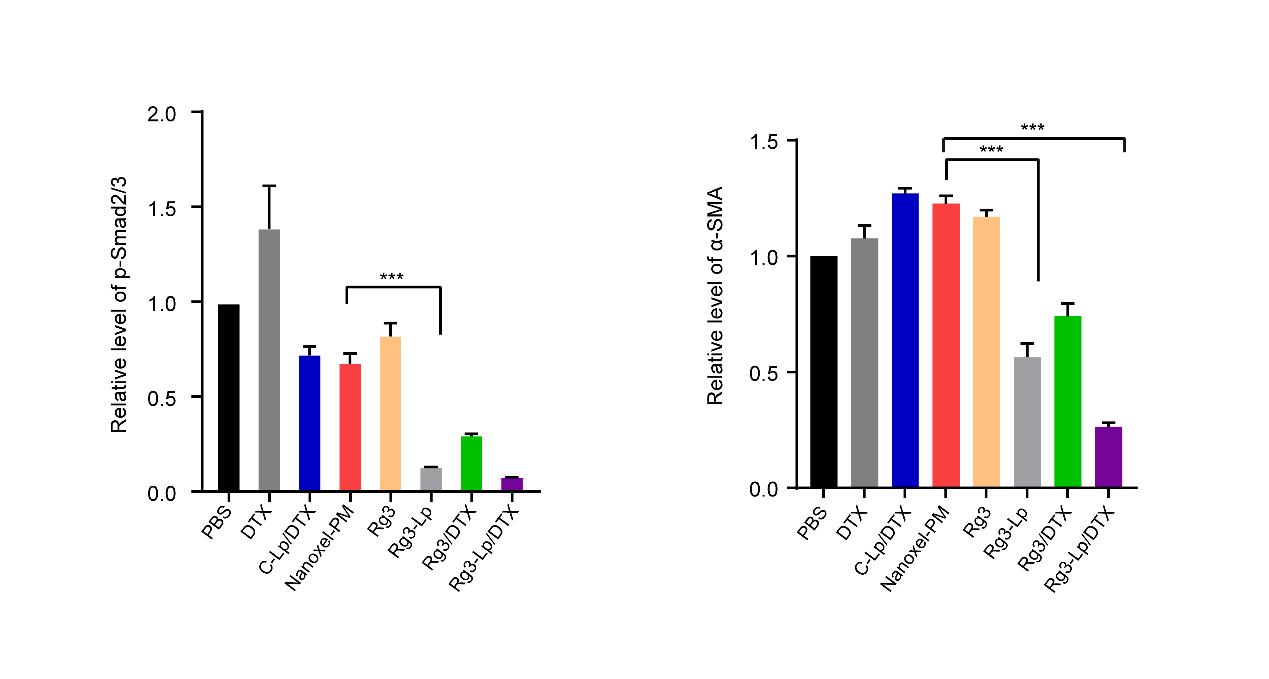


**Figure S10.** Quantitative western blot analysis of the level of p-Smad2/3 and α-SMA in tumor tissues after different treatments (n=3).


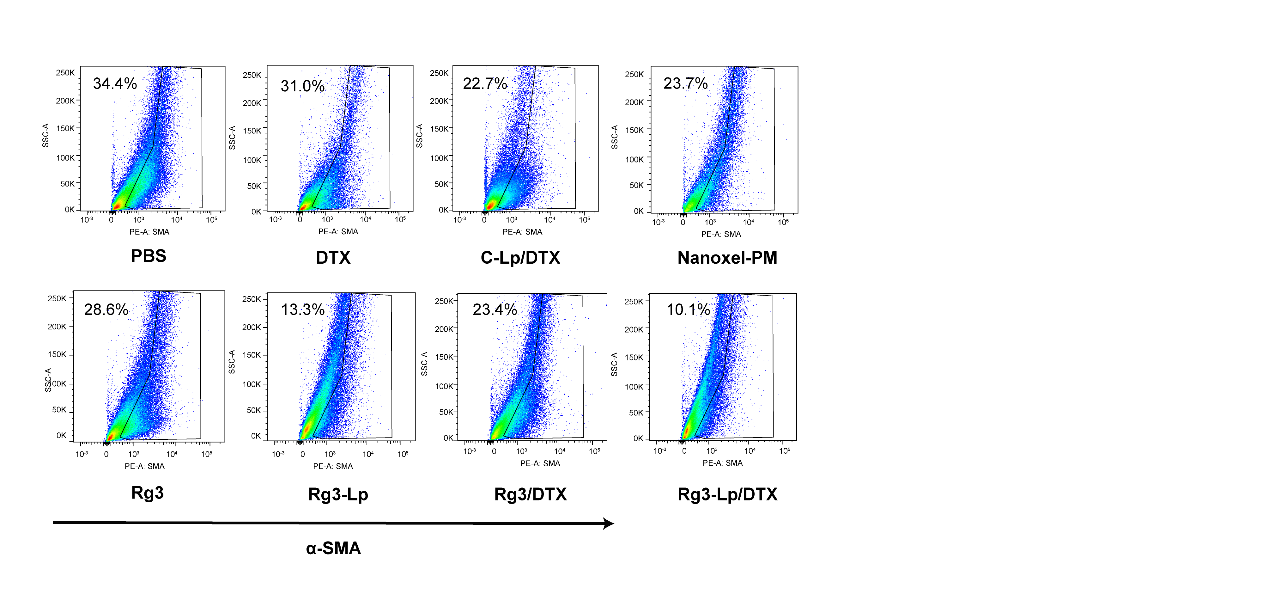


**Figure S11.** Flow cytometry analysis of activated CAFs in tumor after treatment with PBS, DTX, C-Lp/DTX, Nanoxel-PM, Rg3, Rg3-Lp, Rg3/DTX and Rg3-Lp/DTX, respectively.


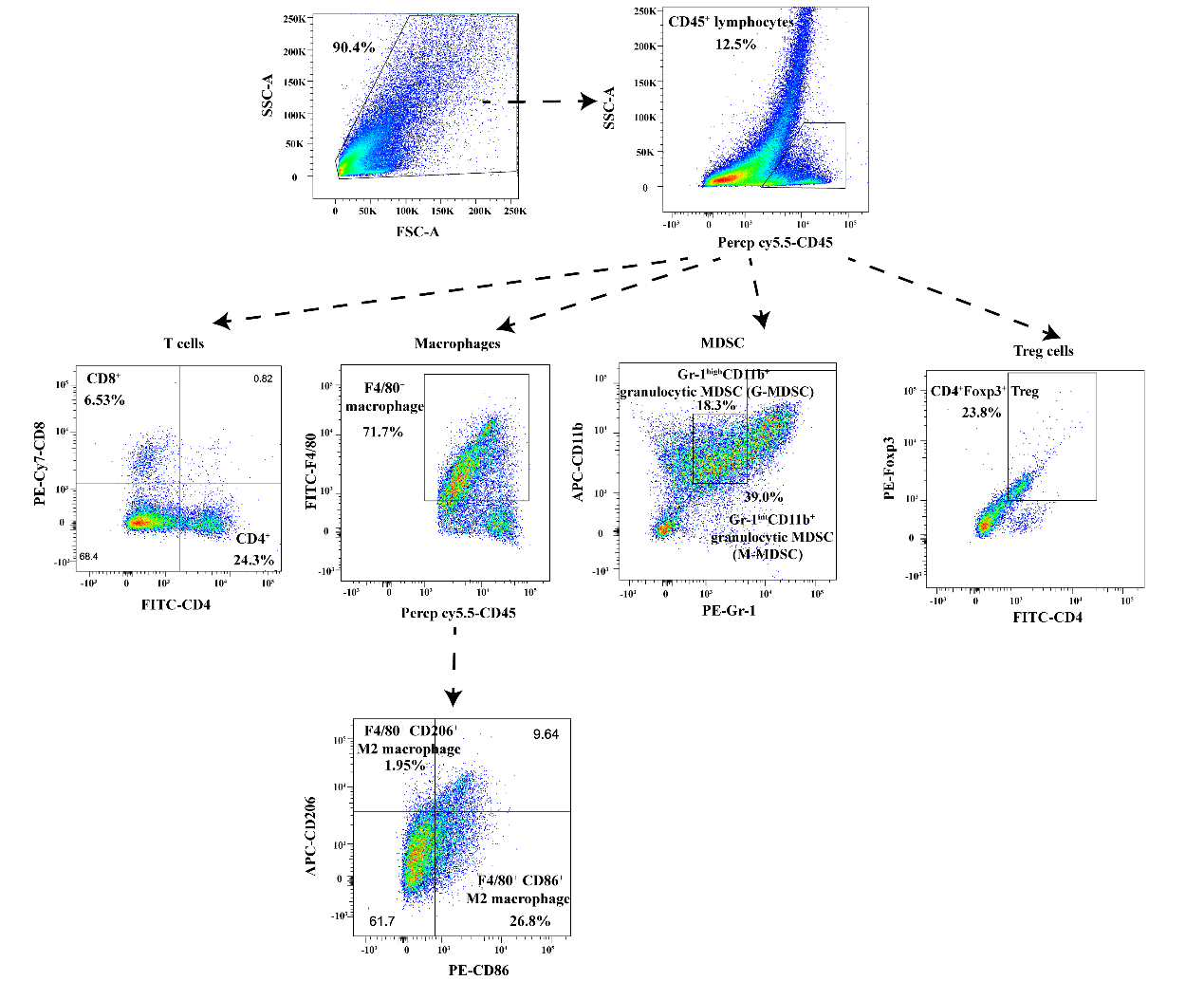


**Figure** **S12**. Representative gating strategy used for flow cytometry analysis of CD4^+^ T cells, CD8^+^ T cells, MDSC (CD11b^+^Gr1^+^), M1 macrophages (F4/80^+^CD86^+^), M2 macrophages (F4/80^+^CD206^+^), and Treg cells (CD4^+^Foxp3^+^) in the tumors (gated on CD45^+^ cells) after different treatments.


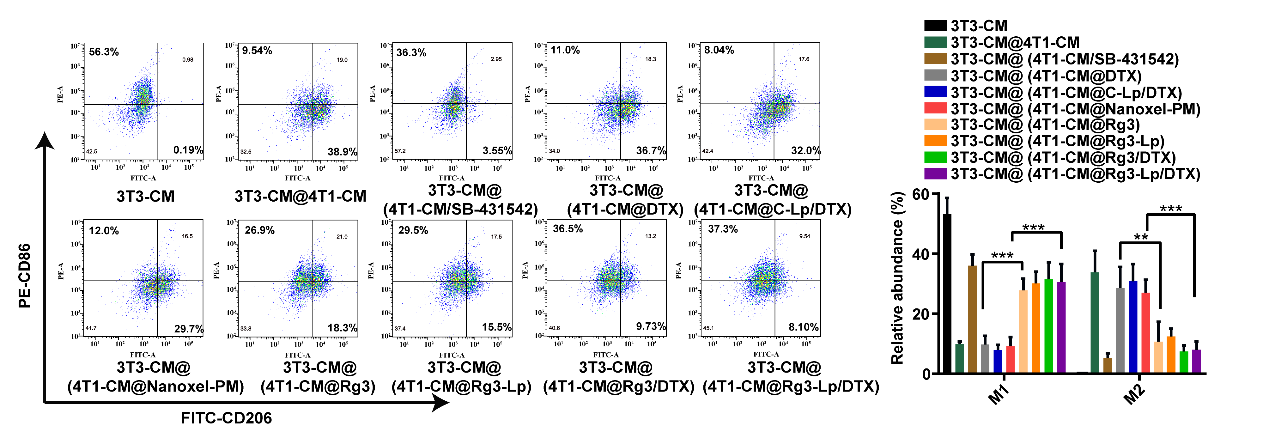


**Figure S13.** Flow cytometric and histogram analysis of M1-type and M2-type macrophages after different treatment (n=3).


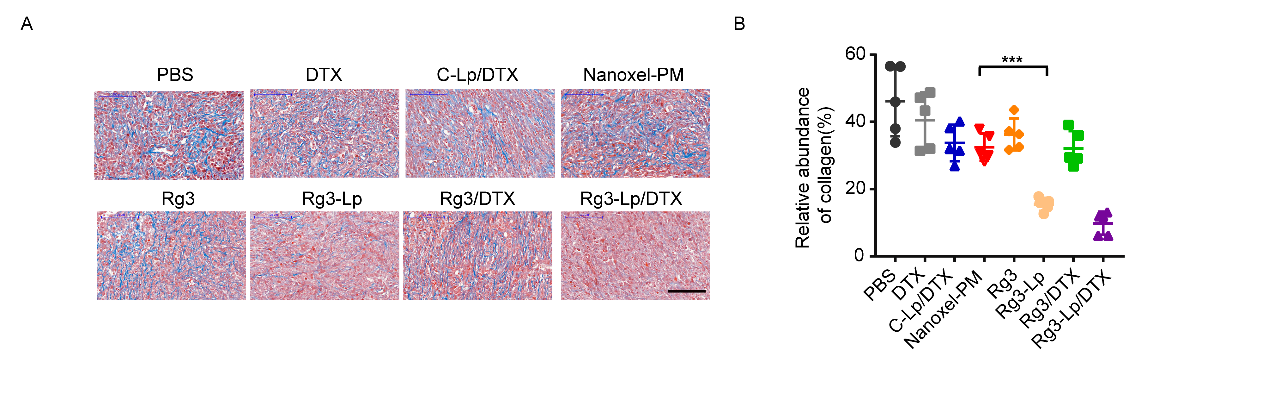


**Figure S14.** Analysis of the level of collagen in tumor tissues measured by Masson’s trichrome staining. (A) Qualitative analysis of the level of collagens in tumor slices measured by Masson’s trichrome staining (blue) (Scale bar 100 μm). (B)Semi-quantitative analysis of the collagens in tumor slices treated with PBS and different DTX formulations. n=3 in each group.


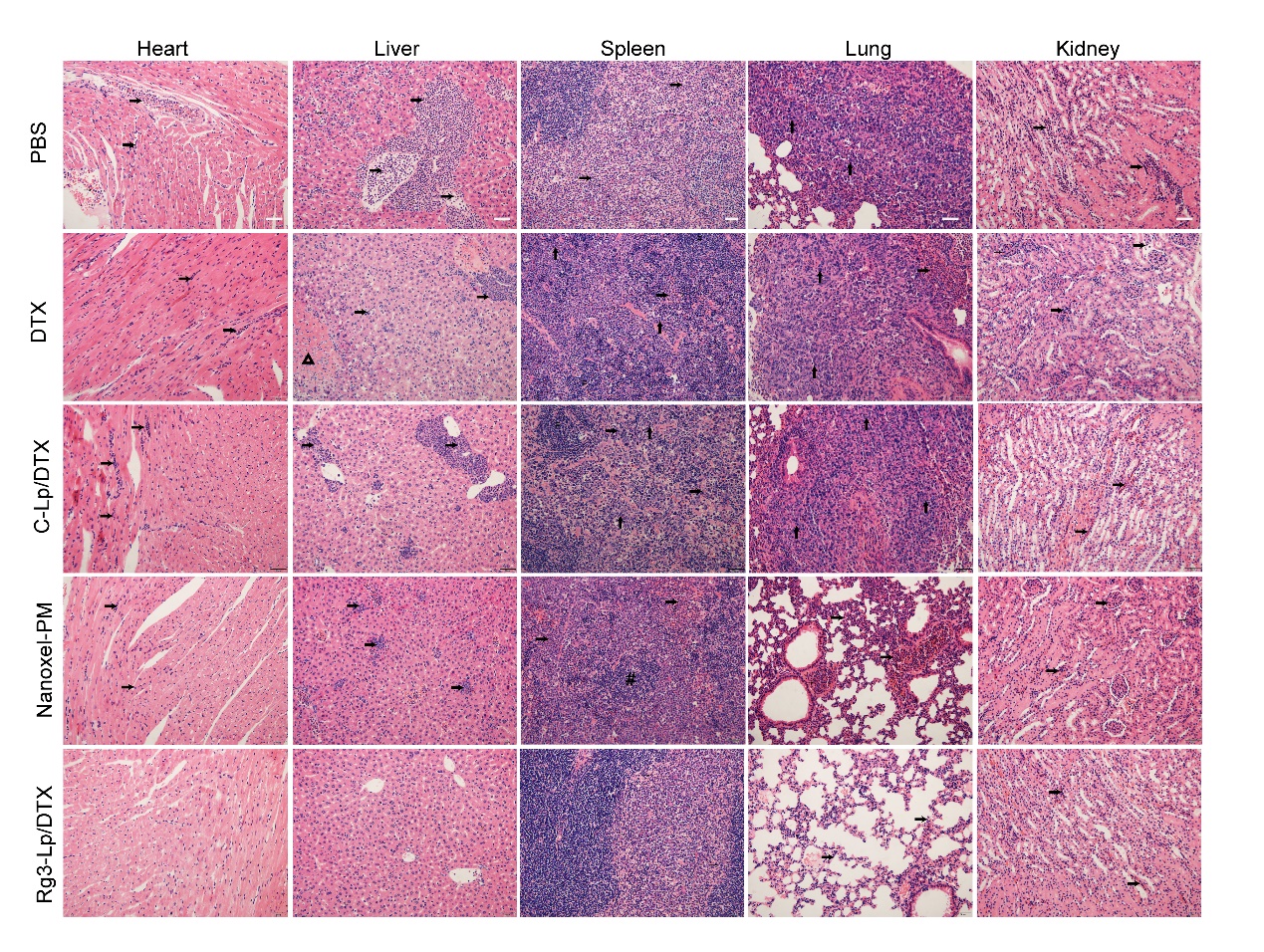


**Figure S15.** H&E staining of major organs. Histopathological analysis of tissue sections stained with Hematoxylin and Eosin after treatments with PBS and different DTX formulations. The scale bar is 50 mm. The symbol “→”, “↑”, “△” and “#” represents the inflammatory site, metastasis site, necrotic site and white pulp decreased site respectively.
